# Full-Spectrum-Responsive Au@Cu_7_S_4_‑Decorated Monoclinic TiO_2_ Nanowires for Solar Hydrogen Production

**DOI:** 10.1021/acsami.5c03462

**Published:** 2025-06-17

**Authors:** Yu-Ting Wang, Hsuan-Hung Kuo, Chun-Yi Chen, Tso-Fu Mark Chang, Masato Sone, Yung-Jung Hsu

**Affiliations:** † Department of Materials Science and Engineering, 34914 National Yang Ming Chiao Tung University, Hsinchu 300093, Taiwan; ‡ Institute of Integrated Research, Institute of Science Tokyo, Kanagawa 226-8503, Japan; § Center for Emergent Functional Matter Science, National Yang Ming Chiao Tung University, Hsinchu 300093, Taiwan

**Keywords:** monoclinic TiO_2_, plasmonic, near-infrared-responsive, solar hydrogen production, Z-scheme

## Abstract

Developing photocatalysts that can efficiently capture light across a broad spectrum, from ultraviolet to near-infrared, is crucial for maximizing solar energy utilization. Such broad-spectrum responsiveness enhances solar energy utilization in photocatalysis, enabling a more sustainable and efficient pathway for hydrogen production. The limited availability of photocatalysts capable of responding to near-infrared irradiation underscores the urgent need for the development of versatile near-infrared-responsive photocatalysts. In this work, TiO_2_ nanowires in the monoclinic phase, a less common crystallographic form of TiO_2_, were synthesized, followed by a decoration with Au particles surrounded by a hollow Cu_7_S_4_ shell. The resulting TiO_2_-Au@Cu_7_S_4_ heterostructure nanowires exhibited remarkable properties conducive to efficient solar hydrogen production. The band structure alignment among TiO_2_, Au, and Cu_7_S_4_ induced a Z-scheme charge separation mechanism, which boosted both the carrier utilization efficiency and redox powers. Furthermore, incorporating Au@Cu_7_S_4_ significantly broadened the absorption capability of TiO_2_ into the visible and near-infrared spectral ranges. This enhancement arose primarily from the inherent bandgap absorption of Cu_7_S_4_, as well as the plasmonic properties of Au and Cu_7_S_4_ components. Additionally, the hydrophilic surface of TiO_2_-Au@Cu_7_S_4_ enhanced the water accessibility, which promoted interactions between water molecules and the photocatalyst surface. By integrating these characteristics, TiO_2_-Au@Cu_7_S_4_ heterostructure nanowires demonstrated noteworthy efficiency in solar hydrogen production across a wide spectral region, achieving notable apparent quantum yields of 10.51% at 300 nm, 4.38% at 450 nm, 4.17% at 800 nm, and 3.66% at 1800 nm. Notably, TiO_2_-Au@Cu_7_S_4_ surpassed all of the near-infrared-responsive TiO_2_-based photocatalysts ever reported in hydrogen production. The findings can provide a practical strategy to design a full-spectrum-responsive TiO_2_-based photocatalyst for widespread use in photocatalytic processes.

## Graphical abstract

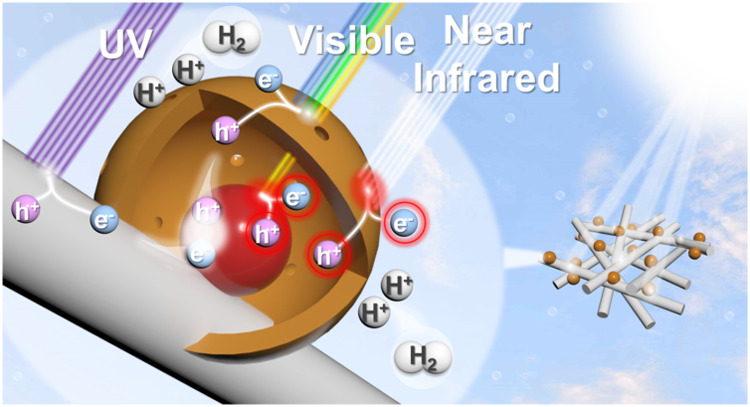

## Introduction

Driven by the growing demand for environmental remediation and sustainable energy, semiconductor-based photocatalysis has garnered significant research interest in the last two decades. Identifying a photocatalytic platform that is both robust and practically efficient remains a key challenge. Acknowledged for its superior photocatalytic characteristics, TiO_2_ stands out as a top-performing material with substantial chemical resilience, low toxicity, and versatility across a broad range of applications. It has found wide application in various areas, such as degradation of water splitting,^1^ environmental pollutants,^2^ organic transformation,^3^ and carbon dioxide reduction.^4^ Although significant advancements have been made, TiO_2_-based photocatalysis continues to face key challenges. TiO_2_ inherently possesses a large bandgap, typically reported as approximately 3.20 eV for anatase, 3.00 eV for rutile, and 3.30 eV for brookite phases.^5^ This broad bandgap limits its optical absorption predominantly to the ultraviolet (UV) region, a relatively small fraction of entire solar spectrum. To enable light harvesting across the visible range and enhance carrier utilization, researchers have explored surface modifications and band structure engineering. A widely adopted strategy involves integrating TiO_2_ with narrow-bandgap semiconductors that possess favorable band alignment. Examples of typical designs include p–n heterojunctions formed by CuO-TiO_2_,^6^ type-II composites, such as TiO_2_-CdS core–shell structures,^7^ and Z-scheme TiO_2_-Au-CdS systems.^8^ These features collectively enhance visible photon harvesting and enable efficient carrier separation, ultimately resulting in a substantial improvement in the photocatalytic activity. Furthermore, the inclusion of plasmonic metals like Au,^9^ Ag,^10^ and Pt/SiO_2_ ^11^ further improves the photoresponse of TiO_2_. This enhancement primarily arises from the localized surface plasmon resonance (LSPR) effect, which boosts the formation of charge carriers and strengthens local electric fields, thereby significantly elevating the photocatalytic performance.

While considerable advances have been made in designing TiO_2_-based heterostructures for visible light response, efforts toward near-infrared-responsive TiO_2_ photocatalysts remain relatively scarce. Solar radiation is composed of approximately 7 % UV (λ < 400 nm), 39 % visible (400–700 nm), and 54 % near-infrared (700–3000 nm) spectral regions. It is important to note that the largely underused near-infrared portion represents a significant source of solar energy that remains largely untapped. Thus, the advancement of photocatalysts capable of utilizing near-infrared light is considered a critical step toward realizing full-spectrum solar-driven hydrogen production. Incorporation of noble metal nanostructures, particularly those exhibiting LSPR, has been explored as a strategy to harness near-infrared photons. Plasmonic metals, for example, Au,^12^ Ag,^13^ and Cu,^14^ have been widely studied for this purpose. Among them, Au nanostructures are especially promising due to their tunable LSPR absorption, which can extend from the visible to the near-infrared region. This spectral tunability provides an effective strategy for utilizing longer-wavelength light, where conventional photocatalysts exhibit limited performance. Recently, nonstoichiometric semiconductors with intrinsic defects have emerged as promising materials for near-infrared-responsive photocatalysis. Examples include p-type Cu_2–*x*_S^15^ and Cu_2–*x*_Se,^16^ in addition to n-type WO_3–*x*_ ^17^ and MoO_3–*x*_,^18^ all of which absorb near-infrared photons owing to LSPR. This characteristic originates from abundant charge carriers, which typically form because of intrinsic defects, such as vacancies of Cu or O.^19–21^ In contrast to conventional metals, where LSPR arises from collective oscillation of free electrons, LSPR in these nonstoichiometric semiconductors is induced by an excess of charge carriers, which is a result of stoichiometric imbalances. For example, Cu vacancies in Cu_2–*x*_S and Cu_2–*x*_Se generate free holes, whereas oxygen vacancies in WO_3–*x*_ and MoO_3–*x*_ lead to the formation of electrons. A significant benefit offered by these materials is the ability to finely adjust their LSPR frequency via controlled variation in nonstoichiometry or doping, a characteristic typically not achievable in noble metals.^19^

TiO_2_ occurs naturally in various crystalline structures, among which anatase, rutile, and brookite are the most prominent. The selection of a particular crystalline phase for TiO_2_ photocatalysts typically depends on the unique requirements of the intended application. Both anatase and rutile TiO_2_ have found extensive utilization in photocatalytic applications. It has been observed that TiO_2_ in a mixed-phase configuration, especially with both anatase and rutile present, demonstrates enhanced photocatalytic activity in comparison to its individual polymorphs.^22^ Brookite TiO_2_ has demonstrated superior photocatalytic efficiency to anatase and rutile phases by virtue of the longer electron lifetime.^23^ Monoclinic phase of TiO_2_, on the other hand, represents a relatively rare crystallographic structure that has garnered increasing attention for its distinct physical and chemical behavior. The potential applications of monoclinic TiO_2_ in photocatalysis are gaining interest due to unique properties that distinguish it from the other three traditional phases.^24,25^ Notably, monoclinic TiO_2_ has been reported to possess a conduction band level that is more negative than those of anatase and rutile,^26^ making it more suited for conducting photocatalytic reduction reactions, such as hydrogen generation,^27–29^ reduction of carbon dioxide,^30,31^ as well as nitrogen fixation.^32^ Moreover, monoclinic TiO_2_ has a relatively smaller bandgap (*E*_g_ = 3.0 eV),^33^ rendering a higher capacity for harvesting photons from sunlight, which is advantageous to enhancing the overall photocatalytic performance. These features have made monoclinic TiO_2_ particularly interesting as a new photocatalyst model system with tailored photocatalytic properties.

In this work, monoclinic TiO_2_ nanowires were synthesized, followed by decoration with Au particles surrounded by a hollow Cu_7_S_4_ shell, creating a Z-scheme heterostructure. Note that to achieve pronounced charge separation for TiO_2_, heterostructures based on p–n, type-II, and Z-scheme charge transfer models have been proposed and realized. Among them, the Z-scheme mechanism is particularly appealing because it can endow remarkable charge separation and retain high redox powers for the separated charge carriers. To facilitate the Z-scheme carrier transfer mechanism, noble metal particles are commonly employed as electron mediators. These metallic components serve as bridges that enable directional electron flow between two adjacent semiconductors, securing interfacial electron migration and promoting the spatial separation of charges.^34,35^ In the present study, a Z-scheme heterostructure was constructed by sequentially depositing a Cu_2_O layer onto Au-decorated monoclinic TiO_2_ nanowires, followed by a sulfidation process to convert Cu_2_O into Cu_7_S_4_. Owing to the nanoscale Kirkendall effect during sulfidation,^36^ abundant voids were generated and merged, giving rise to the creation of a hollow Cu_7_S_4_ shell encapsulating the Au particles. The resulting TiO_2_-Au@Cu_7_S_4_ heterostructure nanowires exhibited remarkable properties that are advantageous for solar hydrogen production. The specific band alignment among TiO_2_, Au, and Cu_7_S_4_ resulted in a Z-scheme charge transfer pathway, enhancing electron–hole separation, boosting redox capabilities, and consequently leading to improved photocatalytic activity. Additionally, incorporating Au@Cu_7_S_4_ significantly extended the light-harvesting capacity of TiO_2_ into both visible and near-infrared spectral regions, significantly increasing photon absorption and enabling efficient photoactivity under extended spectral irradiation. Furthermore, the hydrophilic surface of TiO_2_-Au@Cu_7_S_4_ enhanced the water accessibility, which promoted interactions between water molecules and the photocatalyst surface. By leveraging these combined attributes, the TiO_2_-Au@Cu_7_S_4_ heterostructure nanowires exhibited efficient solar hydrogen production across a wide spectral region, achieving notable apparent quantum yields (AQYs) of 10.51 % at 300 nm, 4.38 % at 450 nm, 4.17 % at 800 nm, and 3.66 % at 1800 nm.

## Results and Discussion

### Microstructure and Compositions

The detailed procedures for the synthesis and characterization of the samples are given in the Supporting Information.^37–40^ Scanning electron microscopy (SEM) was used to examine the microstructural characteristics. As illustrated in Figure 1(a), pristine TiO_2_ nanowires demonstrated diameters of approximately 30 ± 10 nm and lengths reaching up to 300 ± 100 nm. In Figure 1(b), the TiO_2_-Au sample exhibited a consistent distribution of Au particles on the nanowire surface. Notably, this homogeneous dispersion of Au was essential for achieving the subsequent deposition of Cu_2_O onto the individual Au particles. As displayed in Figure 1(c), upon Cu_2_O deposition, the surface-decorated particles became larger without aggregation. Further sulfidation treatment led to an obvious growth in size for the surface-decorated particles, which was illustrated in Figure 1(d). Figure 1(e) displays an X-ray diffraction (XRD) profile for pristine TiO_2_, showing a diffraction pattern matching that expected for the monoclinic crystallographic structure. For the three heterostructure nanowires, the recorded signals were mostly associated with monoclinic TiO_2_ due to its compositional predominance over Au, Cu_2_O, and Cu_7_S_4_ components. This outcome made it improbable to ascertain the crystallographic structures of Au, Cu_2_O, and Cu_7_S_4_ components from the XRD patterns. Nevertheless, to distinguish the crystallographic features of Au, Cu_2_O, and Cu_7_S_4_ components, XRD analyses were also performed on plain Au@Cu_2_O, plain Au@Cu_7_S_4_, pure Cu_2_O, and pure Cu_7_S_4_. As displayed in Figure 1(f), the resultant XRD profiles revealed the existence of fcc Au, cubic Cu_2_O, and monoclinic Cu_7_S_4_ (equal to triclinic Cu_58_S_32_),^41,42^ which was considered as the crystallographic structure of Au, Cu_2_O, and Cu_7_S_4_ components for the three heterostructure nanowires. This inference was substantiated through a detailed microstructural analysis using transmission electron microscopy (TEM).

**Figure 1:**
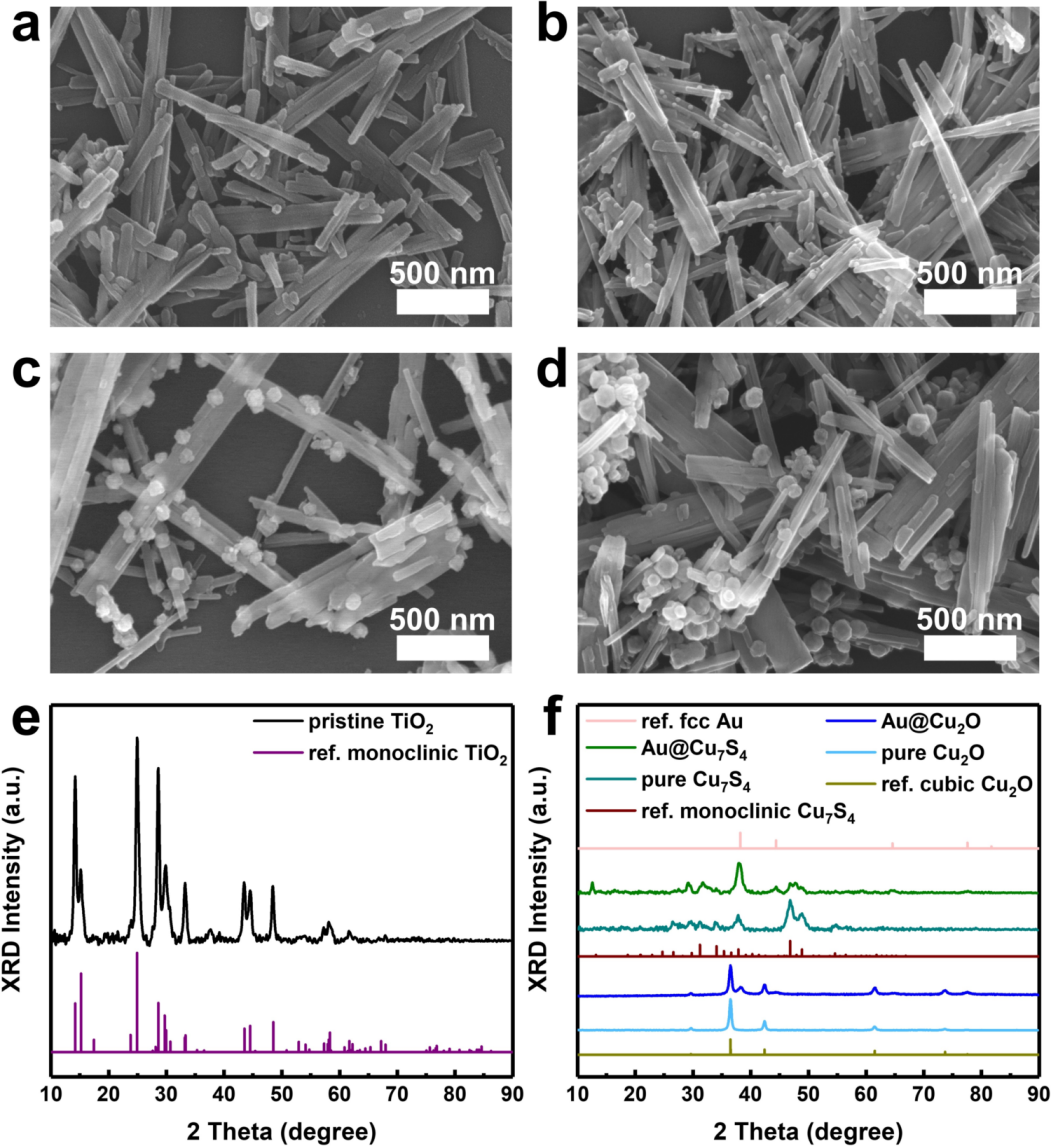
SEM graphs for (a) pristine TiO_2_, (b) TiO_2_-Au, (c) TiO_2_-Au@Cu_2_O, and (d) TiO_2_-Au@Cu_7_S_4_. (e) XRD profiles of pristine TiO_2_ and reference monoclinic TiO_2_ (JCPDS 74–1940). (f) XRD profiles for counterpart samples, reference fcc Au (JCPDS 04–0784), cubic Cu_2_O (JCPDS 01–1172), and monoclinic Cu_7_S_4_ (JCPDS 23–0958).

Figure 2 displays the analytical results of microstructural investigations of the two 3-component heterostructure nanowires. For TiO_2_-Au@Cu_2_O, the surface-decorated Au@Cu_2_O possessed an average core size of 75.8 ± 10.2 nm and an average shell thickness of 60.1 ± 9.5 nm. As displayed in Figure 2(a–c), the measured lattice distances of 0.36, 0.24, and 0.20 nm were attributed to the (110) facet of monoclinic TiO_2_, the (111) facet of fcc Au, and the (111) facet of cubic Cu_2_O, respectively. Additionally, the selected area electron diffraction (SAED) image displayed distinct diffraction rings corresponding to the characteristic planes of monoclinic TiO_2_, fcc Au, and cubic Cu_2_O, which aligned with the XRD analytical results. In Figure 3, energy-dispersive X-ray spectroscopy (EDS) was further used to analyze elemental compositions of Au@Cu_2_O. As present in Figure 3(a), the spatial distributions of Ti, O, Au, and Cu elements for TiO_2_-Au@Cu_2_O confirmed that Au@Cu_2_O core@shell nanocrystals were successfully grown on TiO_2_ nanowires. On the other hand, the TEM, SAED, and EDS analytical results of TiO_2_-Au@Cu_7_S_4_ shown in Figures 2(d,f) and 3(b) disclosed that the surface-decorated Au@Cu_7_S_4_ was composed of an Au core particle surrounded by a hollow Cu_7_S_4_ shell. The size of the Au core particle was 15.6 ± 1.4 nm, whereas the thickness of the Cu_7_S_4_ hollow shell was 9.9 ± 1.8 nm. To highlight the spatial distribution of Cu signals, all of the atomic distribution signals of TiO_2_-Au@Cu_7_S_4_ were integrated into one profile. As shown in Figure S1, the overlapped profile revealed that the distribution of Cu was well aligned with the position of the Cu_7_S_4_ shell of Au@Cu_7_S_4_ surrounding TiO_2_. Notably, the Cu_2_O shell of TiO_2_-Au@Cu_2_O and the Cu_7_S_4_ shell of TiO_2_-Au@Cu_7_S_4_ were not fully compact, showing some degree of porosity. As can be identified from the relatively white image contrast in Figure 2(c,d), both Cu_2_O and Cu_7_S_4_ shells contained microstructural defects such as voids and pores. These defects can provide microscopic channels to enable the movement of molecules from the exterior to the surface of the inner Au. This feature is important to facilitate the occurrence of redox reactions within surface-decorated Au@Cu_2_O and Au@Cu_7_S_4_.

**Figure 2:**
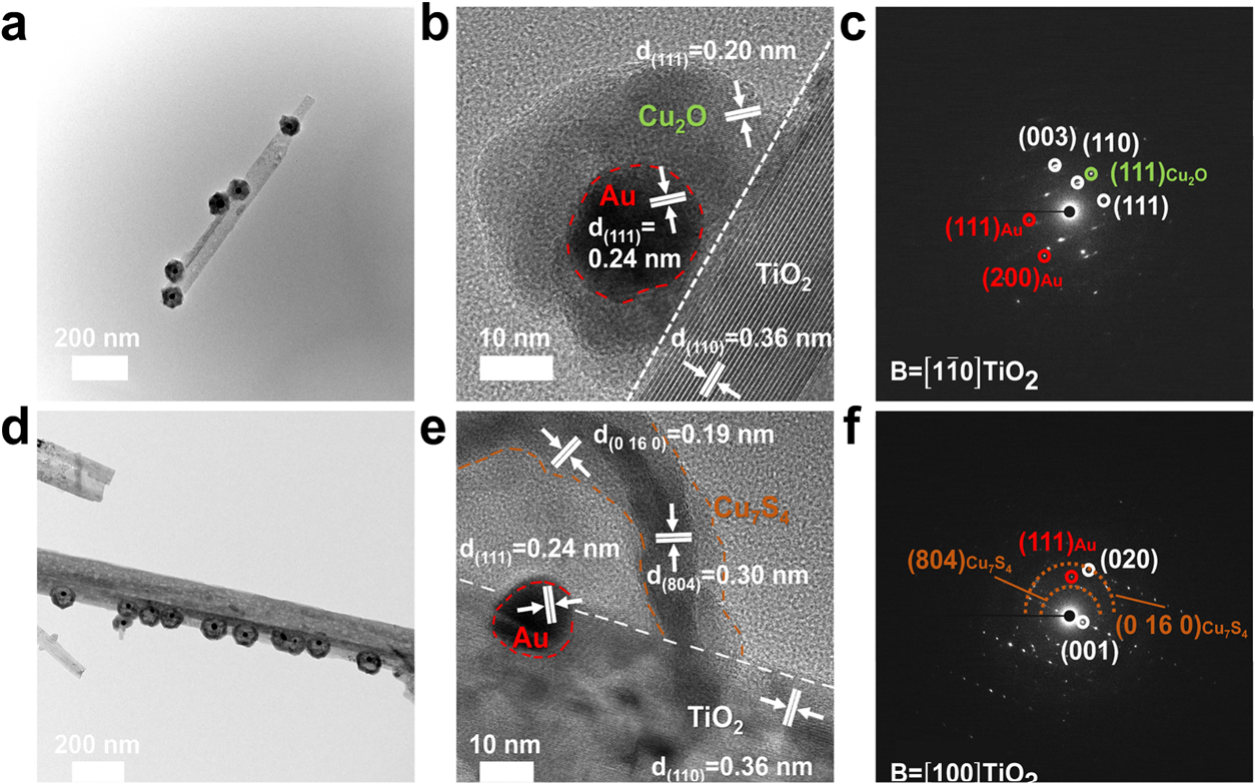
TEM graph, high-resolution TEM graph, and SAED image for (a–c) TiO_2_-Au@Cu_2_O and (d-f) TiO_2_-Au@Cu_7_S_4_.

**Figure 3:**
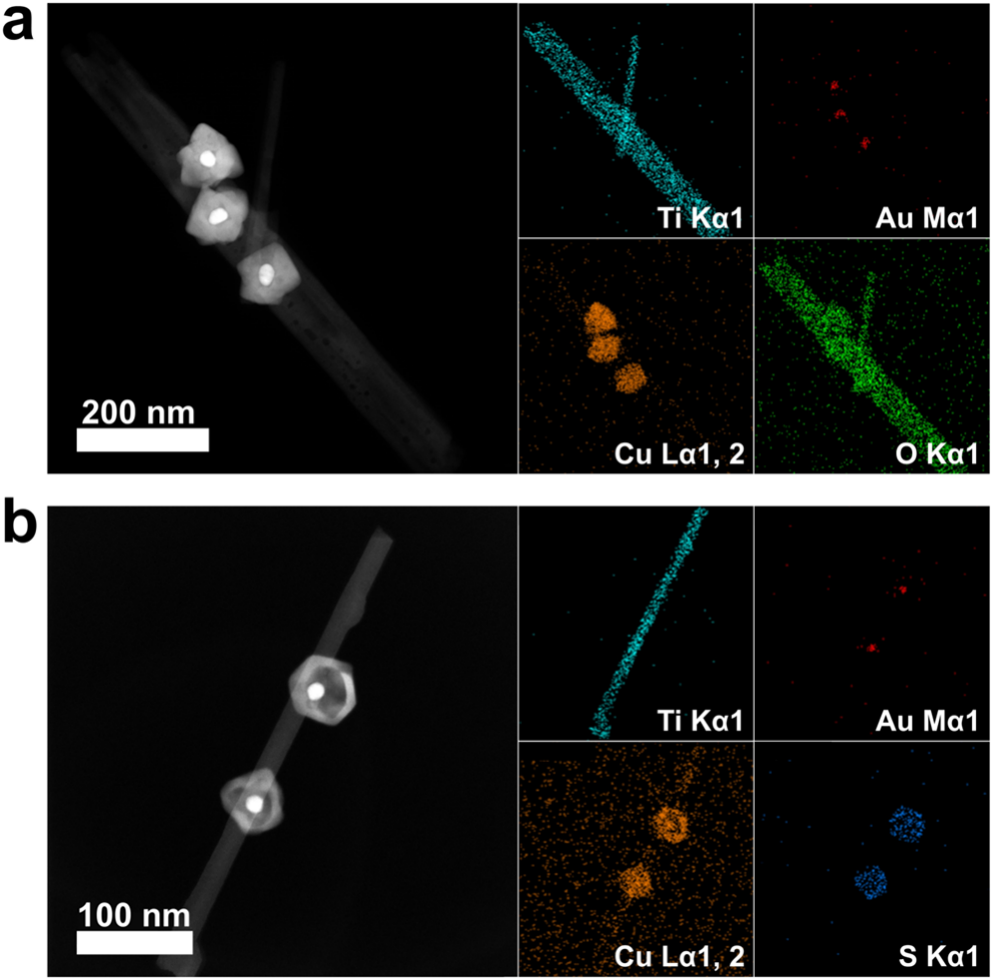
High-angle annular dark-field (HAADF) TEM image with EDS mapping data recorded on (a) TiO_2_-Au@Cu_2_O and (b) TiO_2_-Au@Cu_7_S_4_.

### Interfacial Charge Transfer

The optical properties were characterized with diffuse reflectance spectroscopy (DRS) and photoluminescence (PL) spectroscopic data. As displayed in Figure 4(a), pristine TiO_2_ revealed strong light absorption in the 280–420 nm region, corresponding well with the reported bandgap energy of monoclinic TiO_2_.^43^ For TiO_2_-Au, an additional absorption peak emerged at around 550 nm. The LSPR of Au could account for this absorption.^44^ Upon the deposition of Cu_2_O, the LSPR of Au exhibited a red shift to approximately 650 nm, likely caused by the elevated refractive index of the surrounding Cu_2_O layer (refractive index ranging from 2.8 to 3.4) relative to air (refractive index = 1.0).^45^ In addition to this plasmonic resonance, a distinct absorption edge near 510 nm, attributable to the intrinsic bandgap of Cu_2_O, was also detected.^46^ For TiO_2_-Au@Cu_7_S_4_, besides the bandgap absorption features of TiO_2_ (280–420 nm) and Cu_7_S_4_ (around 600 nm),^47^ another prominent absorption band spanning 600 to 2200 nm was observed. This broad absorption might contain signals from the LSPR of Au and the LSPR of Cu_7_S_4_,^21^ which is significant concerning the effective harvesting of the solar spectrum in the near-infrared region for photocatalytic applications. Figure 4(b) further shows the DRS spectra for the four counterpart samples, which possessed absorption characteristics consistent with those of the two 3-component heterostructure nanowires. Au@Cu_2_O exhibited two distinct optical features. A broad absorption peak centered at around 655 nm was associated with the LSPR of Au. In addition to this plasmonic response, clear absorption across the UV to visible region was detected. A comparable edge, linked to the intrinsic bandgap of Cu_2_O, was also observed in the spectrum of pure Cu_2_O. Similarly, Au@Cu_7_S_4_ and pure Cu_7_S_4_ demonstrated identical characteristic absorption behavior, including a sharp edge reaching into the visible range and a wide absorption band extending into the near-infrared spectrum. The bandgap energy of TiO_2_, Cu_2_O, and Cu_7_S_4_ components was further determined by analyzing the corresponding Tauc plots of pristine TiO_2_, pure Cu_2_O, and pure Cu_7_S_4_. As shown in Figure S2, the calculated bandgap values of TiO_2_, Cu_2_O, and Cu_7_S_4_ were 3.49, 2.38, and 2.01 eV, respectively.

**Figure 4:**
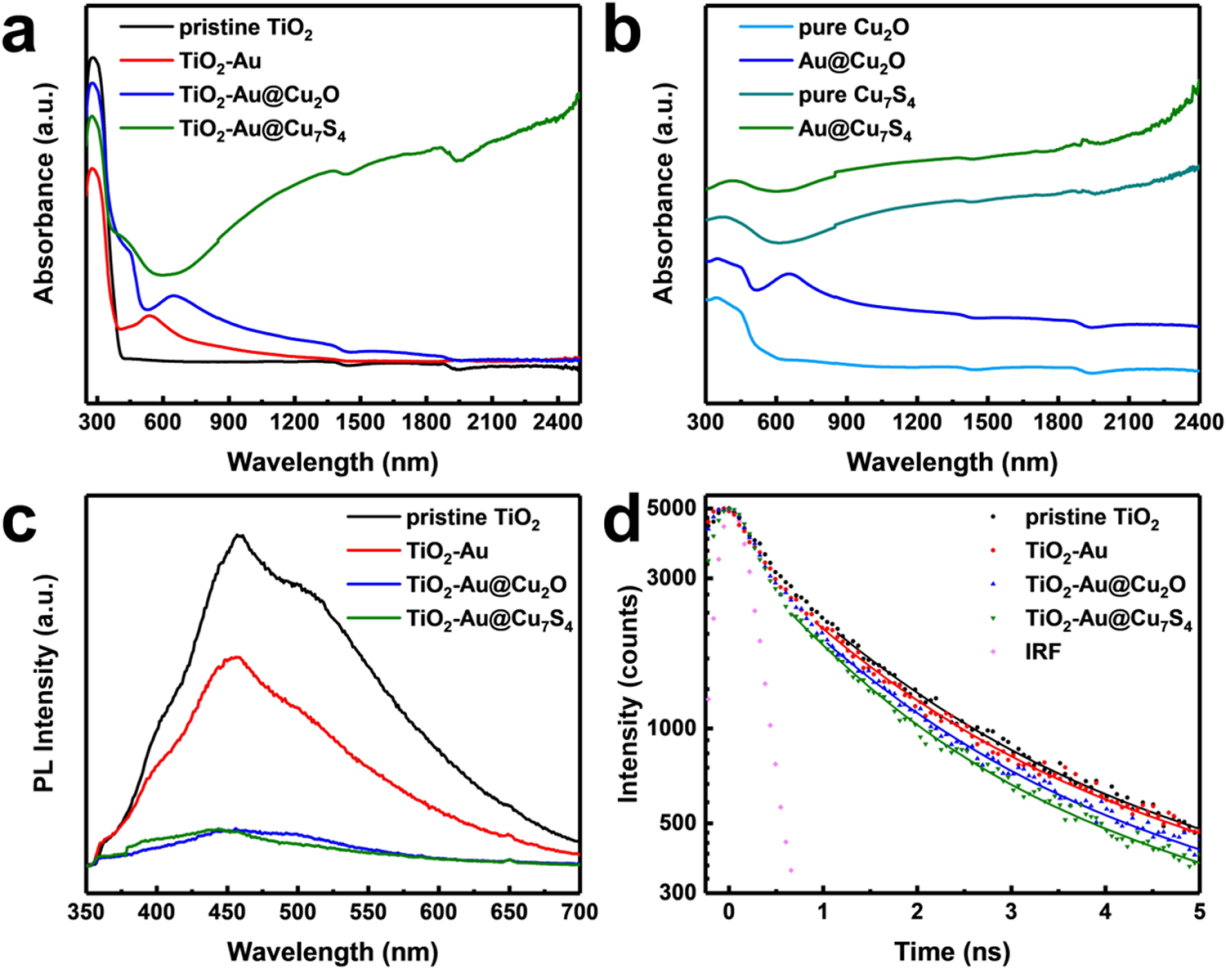
UV–vis-near-infrared DRS spectra of (a) four TiO_2_-based nanowires and (b) four counterpart samples. (c, d) The corresponding steady-state and time-resolved PL profiles.

The present heterostructure nanowire samples served as a suitable platform for investigating how charge transportation processes in TiO_2_ were influenced by the formation of heterostructures. To gain qualitative insights, a steady-state PL spectroscopy analysis was initially performed. In Figure 4(c), pristine TiO_2_ displayed a considerably wide emission band from 350 to 700 nm. If examined closely, three emission bands positioned at 420, 450, and 510 nm can be deconvoluted from the broad emission. The emissions at 420 and 450 nm resulted from electronic transitions at TiO_6_ octahedra involving self-trapped excitons.^48,49^ The emission at 510 nm originated from the trapped electrons from defects or oxygen vacancy states.^50^ TiO_2_-Au exhibited a markedly reduced PL intensity in comparison to that of pristine TiO_2_, suggesting that Au can promote charge separation by reducing charge recombination within TiO_2_. For the two 3-component heterostructure nanowires, the recorded PL intensity was even lower, which indicated that Cu_2_O and Cu_7_S_4_ can further promote charge separation for TiO_2_ by facilitating charge migration across the interface.

Time-resolved PL was used to investigate the interfacial charge dynamics. The collected spectra are shown in Figure 4(d). The three heterostructure nanowires showed faster PL decay kinetics than pristine TiO_2_, reflecting that photoexcited charge carriers of TiO_2_ were preferentially transported to decorated Au, Au@Cu_2_O, and Au@Cu_7_S_4_. The decay curves were interpreted through a biexponential fitting approach, which yielded two separate decay lifetimes and an average lifetime ⟨τ⟩ calculated based on their relative intensities. As detailed in Table S1, the computed PL lifetime of three heterostructure nanowires was shorter than that of pristine TiO_2_. The reduction in the lifetime was ascribed to the transportation of charge carriers from TiO_2_ to the attached particles. Based on this interpretation, the interfacial charge transfer rate constant (*k*_ct_) was calculated according to eq 1:^51^

1 $$k_{\mathrm{ct}}\left({\mathrm{TiO}}_{2}\to P\right)=\frac{1}{\left\langle \tau \right\rangle }\left({\mathrm{TiO}}_{2}-P\right)-\frac{1}{\left\langle \tau \right\rangle }\left(\mathrm{pristine}\;{\mathrm{TiO}}_{2}\right)$$

In eq 1, *P* denotes the decorated particles. The calculation results showed that *k*_ct_ of 7.2 × 10^6^, 2.42 × 10^7^, and 5.34 × 10^7^ s^–1^ can be, respectively, achieved on TiO_2_-Au, TiO_2_-Au@Cu_2_O, and TiO_2_-Au@Cu_7_S_4_. Among the three heterostructure nanowires, TiO_2_-Au@Cu_7_S_4_ had the highest *k*_ct_. This outcome signified that TiO_2_-Au@Cu_7_S_4_ attained the highest charge separation efficiency. It should be emphasized that steady-state PL data are an outcome of charge transfer for a substantially long time. Under this regime, charge migration from TiO_2_ to the decorated Au@Cu_2_O and Au@Cu_7_S_4_ would reach a saturation state, which further led to a comparable extent of PL intensity depression for them.

### Band Alignment and Charge Transfer Mechanism

The band alignment at the interface was explored by X-ray photoelectron spectroscopy (XPS) and ultraviolet photoelectron spectroscopy (UPS). Figure S3 presents the Ti 2p and O 1s spectra collected on four samples. In Figure S3­(a), the Ti 2p spectrum of pristine TiO_2_ displayed a distinct doublet with peaks located at around 457.5 eV (Ti 2p_3/2_) and 467.3 eV (Ti 2p_1/2_). The binding energies of Ti 2p doublets were noticeably enlarged for the three heterostructure nanowires, which were 458.0 eV (Ti 2p_3/2_) and 463.6 eV (Ti 2p_1/2_) for TiO_2_-Au, 457.9 eV (Ti 2p_3/2_) and 463.5 eV (Ti 2p_1/2_) for TiO_2_-Au@Cu_2_O, and 458.2 eV (Ti 2p_3/2_) and 463.8 eV (Ti 2p_1/2_) for TiO_2_-Au@Cu_7_S_4_. The conspicuous electronic interactions between TiO_2_ and the surface-decorated particles can give rise to such binding energy shifts.^52,53^ Figure S3­(b) presents the corresponding XPS spectra of O 1s. Pristine TiO_2_ showed deconvoluted peaks appearing at 528.8 and 531.0 eV, which resulted from Ti–O^54^ and O–H bonding,^55^ respectively. As for the three heterostructure nanowires, the deconvoluted peaks associated with Ti–O and O–H bonding were shifted to 529.1 and 531.3 eV (TiO_2_-Au), 529.1 and 530.4 eV (TiO_2_-Au@Cu_2_O), and 529.4 and 531.4 eV (TiO_2_-Au@Cu_7_S_4_). Moreover, TiO_2_-Au@Cu_2_O exhibited an additional peak at 532.9 eV related to the absorbed water on Cu_2_O.^56^ The Au 4f, Cu 2p, and S 2p XPS data for TiO_2_-Au@Cu_7_S_4_ were also investigated in Figure S4. The Au 4f doublets were absent, likely due to the thick Cu_7_S_4_ shell (approximately 10 nm) surrounding the Au yolk, which hindered the escape of the photoelectrons. The Cu 2p and S 2p doublets, on the other hand, showed binding energies consistent with those observed in plain Au@Cu_7_S_4_.^15^

To further interpret the electronic interactions at the interface, UPS measurements were conducted to determine the band structure of each component. Figure S5­(a–c) displays the UPS spectra for pristine TiO_2_, pure Cu_2_O, and pure Cu_7_S_4_ in the region near 0 eV. The spectrum near 0 eV provides quantitative insight into the energy difference between the valence band level (*E*_VB_) and Fermi level (*E*_F_). By linear extrapolation of the tangent to the onset, the *E*_VB_ relative to *E*_F_ was estimated to be around 2.88 eV for pristine TiO_2_, 0.56 eV for pure Cu_2_O, and 0.34 eV for pure Cu_7_S_4_. Figure S5­(d) shows the corresponding UPS spectra in the high-energy region. For pristine TiO_2_, the high-energy spectrum indicated a cutoff energy of 16.58 eV. The energy of incident photon (21.22 eV) minus this cutoff value left an apparent work function, which was calculated as 4.64 eV for pristine TiO_2_. Adding this value to the energy difference obtained in Figure S5­(a), the *E*_VB_ of pristine TiO_2_ was estimated as −7.52 eV. The conduction band level (*E*_CB_) can then be estimated as −4.03 eV by summing up the optical bandgap. Following the same method, the apparent work function for pure Au, pure Cu_2_O, and pure Cu_7_S_4_ was also calculated from Figure S5­(d), respectively, giving *E*_F_ values of −4.34, −4.75, and −4.20 eV. Similarly, the *E*_VB_ and *E*_CB_ values of pure Cu_2_O and pure Cu_7_S_4_ can be, respectively, estimated as −5.31 and −2.93 eV for pure Cu_2_O, and −4.54 and −2.53 eV for pure Cu_7_S_4_. Using these energy levels, possible band alignments at the interface of the three heterostructure nanowires are proposed in Figure 5. For TiO_2_-Au, the higher *E*_F_ of Au resulted in downward band bending of TiO_2_. Under light illumination, photogenerated electrons in TiO_2_ tended to transfer to Au along the downward-bent conduction band. The separation of photoexcited electrons from the photogenerated holes may guarantee effective carrier utilization for TiO_2_-Au. For TiO_2_-Au@Cu_2_O, the Fermi level equilibria among the three components led to downward-bent bands for both TiO_2_ and Cu_2_O. These bent bands facilitated the transportation of photoexcited electrons from TiO_2_ and Cu_2_O to Au. The photogenerated holes, on the other hand, tended to migrate from TiO_2_ to Cu_2_O, driven by the relatively higher (more negative) valence band position of TiO_2_ compared to that of Cu_2_O. Such an interfacial carrier transportation greatly suppressed electron–hole recombination within TiO_2_, leading to lower PL intensity with a substantially higher *k*_ct_ than that of TiO_2_-Au. An enhancement in carrier utilization efficiency and thereby a satisfactory photocatalytic activity for TiO_2_-Au@Cu_2_O was expected.

**Figure 5:**
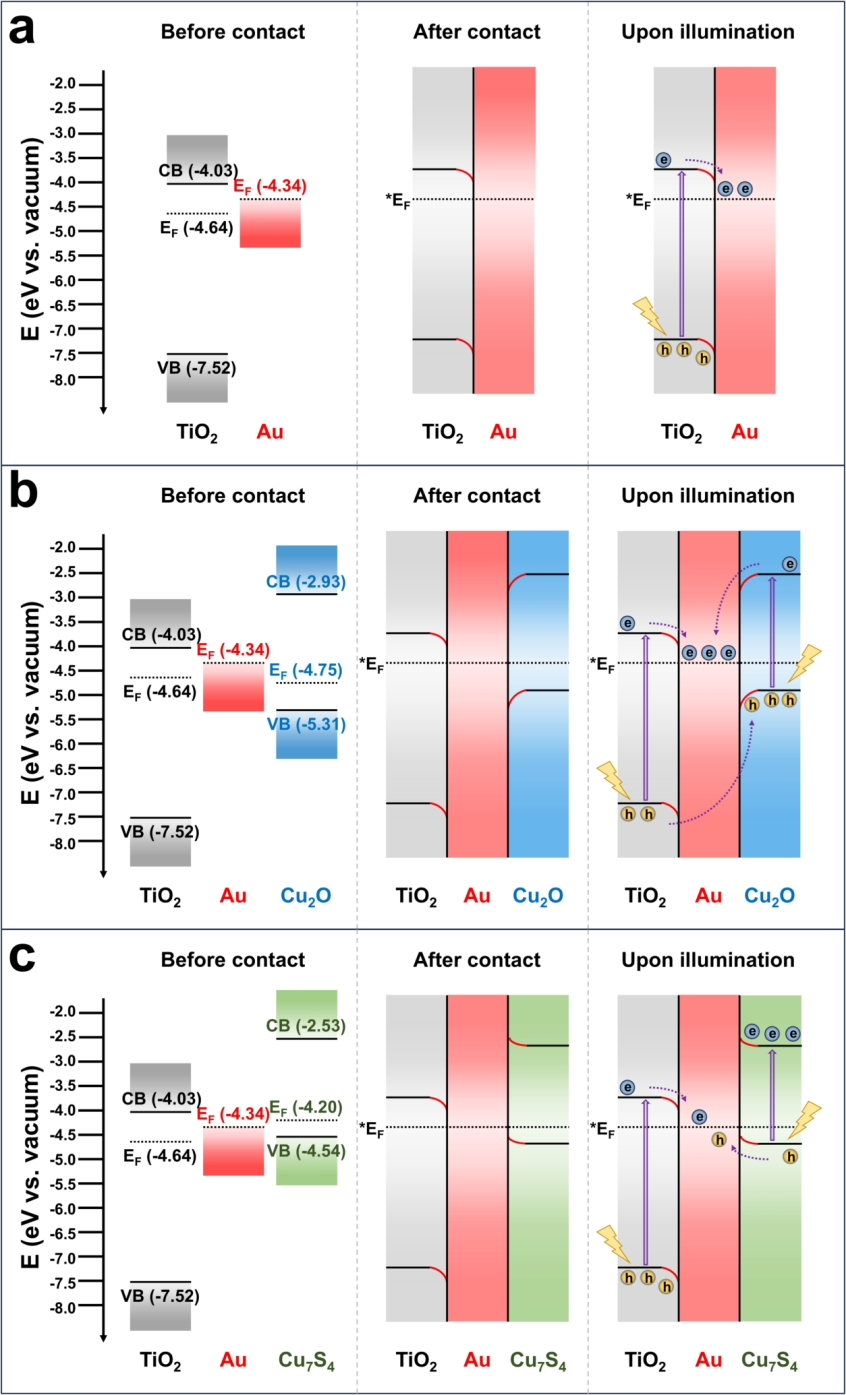
Considered band alignment and charge transfer mechanisms for (a) TiO_2_-Au, (b) TiO_2_-Au@Cu_2_O, and (c) TiO_2_-Au@Cu_7_S_4_.

A different charge transfer scenario was considered for TiO_2_-Au@Cu_7_S_4_. At the Fermi level equilibrium state, the band edges of TiO_2_ and Cu_7_S_4_ were, respectively, downward and upward bent. At the TiO_2_/Au interface, the downward band bending directed electrons from TiO_2_ to Au. At the Au/Cu_7_S_4_ interface, the upward-bent bands encouraged a movement of photogenerated holes from Cu_7_S_4_ to Au. These spatially converged charge carriers within the Au domain became prone to recombination, leading to an accumulation of photoexcited electrons at Cu_7_S_4_ and a concentration of photogenerated holes at TiO_2_. Such a Z-scheme vectorial charge transportation scenario has been widely investigated in various semiconductor/metal/semiconductor heterostructures,^57,58^ proven to be superior to typical metal/semiconductor and type-II semiconductor/semiconductor heterostructures in charge carrier utilization. Importantly, because of the high energetic levels, the accumulated electrons at Cu_7_S_4_ and concentrated holes at TiO_2_ possessed considerably high redox powers. This feature was beneficial for photocatalytic applications, in particular, for solar hydrogen production.

To validate Z-scheme charge transfer, a site-selective photodeposition experiment was conducted.^59–61^ In this experiment, Ag^+^ and Pb^2+^ were, respectively, added to TiO_2_-Au@Cu_7_S_4_ suspension to perform photodeposition of Ag and PbO_2_. By examination of the regions of the deposited Ag and PbO_2_, the reduction site, where the photoexcited electrons were accumulated, and the oxidation site, where the photogenerated holes were concentrated, at TiO_2_-Au@Cu_7_S_4_ can be identified. The photodeposition results for TiO_2_-Au@Cu_7_S_4_ are summarized in Figure 6. Upon the addition of Ag^+^ under light illumination, selective photodeposition of metallic Ag occurred exclusively on the Cu_7_S_4_ domains. This outcome suggested that Cu_7_S_4_ served as the reduction site for Ag^+^ due to the localized accumulation of photoexcited electrons. Conversely, introducing Pb^2+^ led to selective photodeposition of PbO_2_ over the whole TiO_2_ surface. As the photogenerated holes predominantly accumulated on the TiO_2_ domains of TiO_2_-Au@Cu_7_S_4_, the occurrence of the photo-oxidation reaction was localized at TiO_2_, thus selectively forming PbO_2_. Based on the results of selective photodeposition experiments, we concluded that the Z-scheme charge transportation mechanism was prevalent in TiO_2_-Au@Cu_7_S_4_. The proposed charge transfer mechanism for TiO_2_-Au@Cu_2_O was also verified by site-selective photodeposition experiment, and the results are displayed in Figure S6. In the presence of Ag^+^, Ag particles were supposed to selectively form on the Au core of Au@Cu_2_O as photoexcited electrons were primarily concentrated on the Au domain. However, the experimental results presented in Figure S6­(a) showed that Ag particles did not deposit on the Au core. This can be ascribed to the relatively large ionic radius of Ag^+^, which likely hindered its diffusion through the Cu_2_O shell to reach the Au surface. Importantly, Ag particles were not observed at any location on TiO_2_-Au@Cu_2_O, suggesting that photoexcited electrons did not accumulate on TiO_2_ or Cu_2_O. This absence of Ag particle deposition, both on the Au core and elsewhere, provided indirect evidence that the Au core served as the primary electron accumulation location and reduction site. It should be noted that weak Ag signals, randomly distributed across the sampled region, can be observed from TEM-EDS profiles. Although photoexcited electrons were preferentially transported to the Au core, a small fraction may become localized at other sites, particularly once the electron accumulation at the Au core reached saturation. These localized electrons could partially reduce Ag^+^ ions, leading to the weak and scattered Ag signals detected in the TEM-EDS profiles. On the other hand, upon the introduction of Pb^2+^, PbO_2_ was selectively deposited at TiO_2_, which can be noticed from Figure S6­(b). This selective deposition confirmed that photogenerated holes remained within the TiO_2_, identifying it as the primary site for photo-oxidation reactions. Based on the results of selective photodeposition experiment, the proposed charge transfer mechanism of TiO_2_-Au@Cu_2_O can be confirmed.

**Figure 6:**
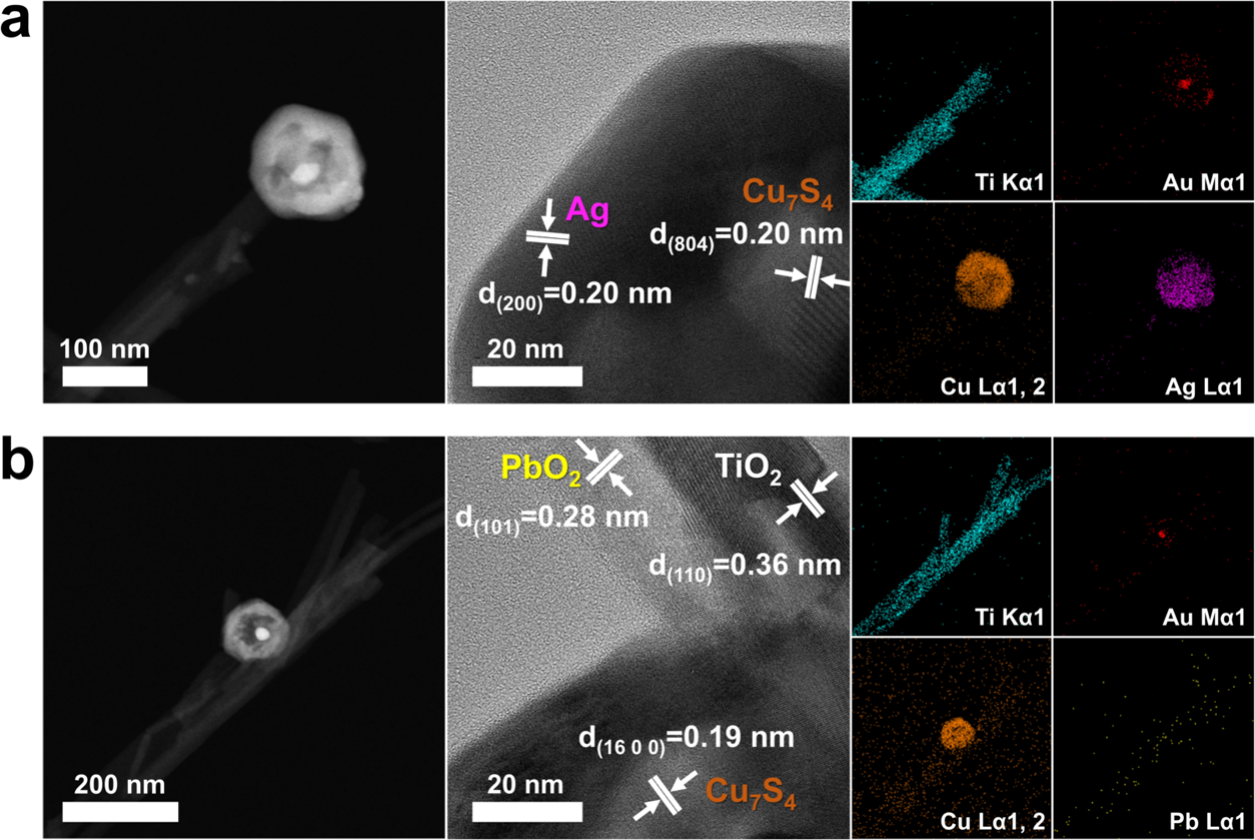
HAADF and HRTEM images, and TEM-EDS mapping profiles for site-selective photodeposition of (a) Ag and (b) PbO_2_ on TiO_2_-Au@Cu_7_S_4_.

### Photocatalytic Hydrogen Production

Figure 7(a) shows the comparative results of hydrogen production among seven sets of samples. Note that error bars were included in the data, which represented the standard deviation from at least three independent measurements. Several important features can be noticed. First, pristine TiO_2_ had a mediocre hydrogen yield of 0.025 μmol h^–1^. This ordinary activity was expected for single-component photocatalysts. Compared to pristine TiO_2_, the three heterostructure nanowire samples all exhibited a noticeable enhancement in hydrogen production, showing a hydrogen yield of 0.046 μmol h^–1^ for TiO_2_-Au, 0.318 μmol h^–1^ for TiO_2_-Au@Cu_2_O, and 0.538 μmol h^–1^ for TiO_2_-Au@Cu_7_S_4_. The improvement in photocatalytic activity was primarily ascribed to more effective charge carrier separation arising from heterostructure creation. It is crucial to highlight that the Au content of TiO_2_-Au can be adjusted to optimize its photocatalytic activity. Figure S7­(a–c) presents SEM graphs for three TiO_2_-Au samples having a progressively higher Au density. Figure S7­(d) compares the hydrogen yields for the three TiO_2_-Au samples. Although all of the TiO_2_-Au samples demonstrated enhanced activity relative to pristine TiO_2_, TiO_2_-Au-3.0 exhibited the highest hydrogen yield among the series. This result suggested the existence of an optimal Au loading that maximized the effectiveness of interfacial charge transfer for activity optimization.^58,62,63^ Second, the three physical mixtures were inferior to the three heterostructure nanowires in hydrogen yield. The recorded hydrogen yields were 0.028 μmol h^–1^ for TiO_2_ + Au, 0.061 μmol h^–1^ for TiO_2_ + Au@Cu_2_O, and 0.187 μmol h^–1^ for TiO_2_ + Au@Cu_7_S_4_. Even though they performed better than pristine TiO_2_, the activity enhancements were much lower than those achieved by the corresponding heterostructure nanowires. This outcome highlighted the necessity of adopting surface decoration to create robust heterostructures on the TiO_2_ surface for optimizing the photocatalytic efficiency. Third, among the three heterostructure nanowires, TiO_2_-Au@Cu_7_S_4_ had the best performance in hydrogen production. This result was consistent with the recorded highest *k*_ct_ from time-resolved PL data. The outstanding hydrogen production performance of TiO_2_-Au@Cu_7_S_4_ emphasized the crucial role of the Z-scheme carrier transportation mechanism, which effectively enhanced carrier utilization and redox potentials, thereby maximizing photocatalytic activity.

**Figure 7:**
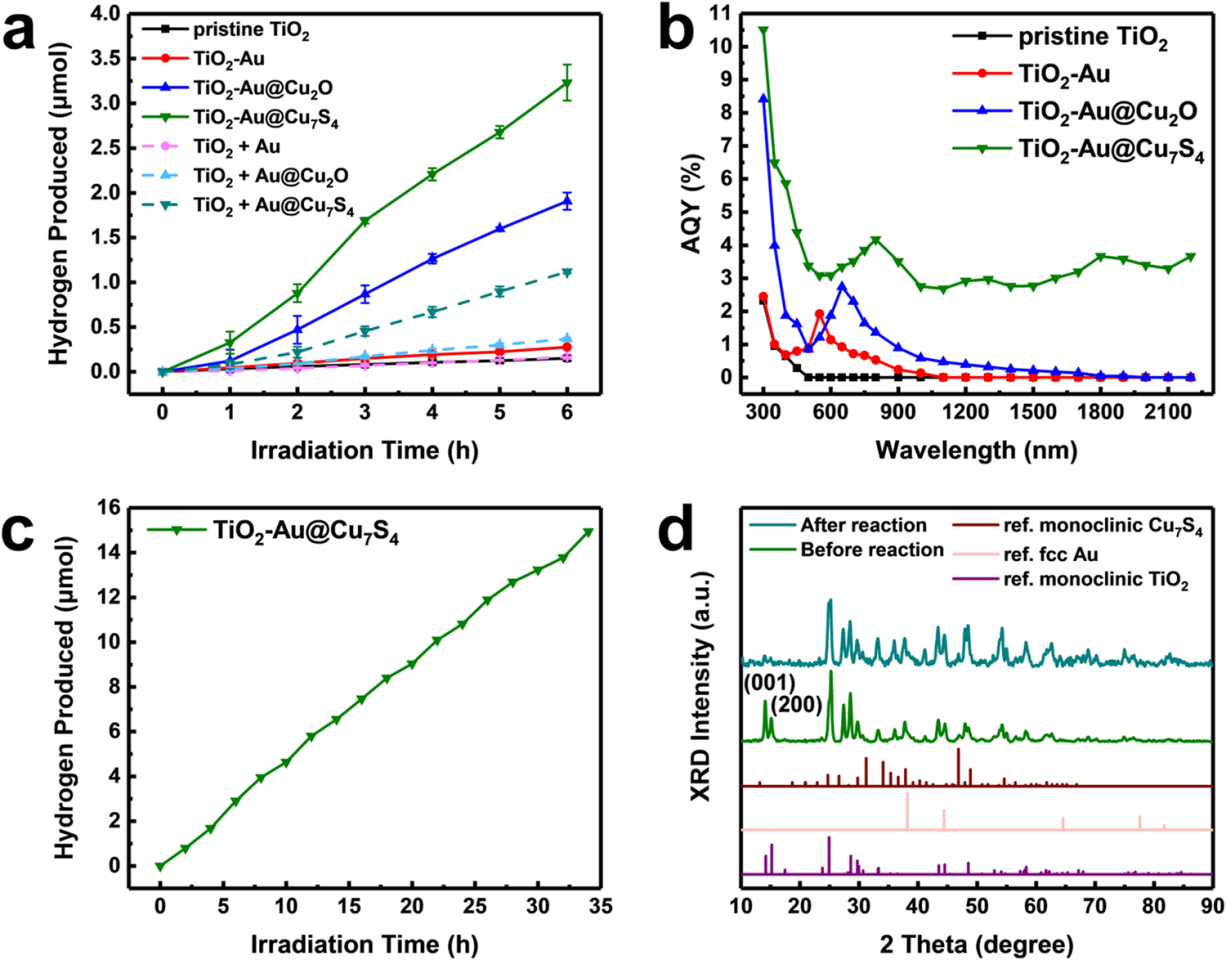
(a) Hydrogen production activity comparison across seven sets of samples. (b) Corresponding AQY spectra of four TiO_2_-based nanowire samples. (c) Long-term stability test of solar hydrogen production on TiO_2_-Au@Cu_7_S_4_. (d) XRD profiles for TiO_2_-Au@Cu_7_S_4_ before and after use in photocatalytic reaction.

To estimate the individual contribution of Cu_7_S_4_, the photocatalytic performance of TiO_2_ + Cu_7_S_4_, a counterpart to TiO_2_-Au@Cu_7_S_4_, was also evaluated. The band alignment of TiO_2_ + Cu_7_S_4_ was studied by using the energy levels measured from UPS. As shown in Figure S8­(a), direct Z-scheme charge transfer was considered for the TiO_2_ + Cu_7_S_4_ system. Upon Fermi level alignment, the band edges of TiO_2_ and Cu_7_S_4_ exhibited downward and upward bending, respectively. The band bending promoted a directional charge transfer, causing electrons to accumulate at Cu_7_S_4_ and holes to concentrate at TiO_2_. Consequently, TiO_2_ + Cu_7_S_4_ was anticipated to exhibit an enhanced photocatalytic performance. As can be seen in Figure S8­(b), TiO_2_ + Cu_7_S_4_ outperformed pristine TiO_2_ in hydrogen yield, underscoring the positive contribution of Cu_7_S_4_ in promoting photocatalytic activity through a direct Z-scheme pathway. However, its performance was considerably inferior to that of TiO_2_-Au@Cu_7_S_4_, highlighting the critical role of Au as an efficient charge transfer mediator in consolidating the effectiveness of Z-scheme mechanism.^43,61^ To assess the impact of various sacrificial agents on photocatalytic activity, a series of commonly used hole scavengers were tested on TiO_2_-Au@Cu_7_S_4_ under identical conditions. Figure S9 shows that 20 % methanol yielded the best hydrogen yield (6.444 μmol h^–1^), followed by 10 % TEOA (3.772 μmol h^–1^) and 20 % ethanol (3.765 μmol h^–1^). In comparison, 0.5 M Na_2_SO_3_ (0.538 μmol h^–1^) and 0.5 M Na_2_SO_4_ (0.173 μmol h^–1^) showed much lower activity, confirming the critical effect of the scavenger type on optimizing photocatalytic performance.

To further understand the activity enhancement for heterostructure nanowires, the hydrogen production activities at different incident wavelengths were compared by using AQYs. Figure 7(b) summarizes the spectral distributions of AQYs for the four TiO_2_-based nanowire samples. These data shared spectral characteristics similar to the corresponding light absorption features revealed from Figure 4(a). The probed photoactivities were thus believed to derive from photon harvesting from incident light. For pristine TiO_2_, the photoactivity was located in the UV region, showing a maximal AQY of 2.32 % at 300 nm. Owing to its large bandgap, the photoactivity of pristine TiO_2_ was limited to the UV region. For TiO_2_-Au, besides the slightly increased photoactivity at the UV region, additional photoactivity was observed at the visible region, showing a peaked AQY of 1.92 % at 550 nm. The incremental photoactivity in the visible region originated from the LSPR effect of Au. For TiO_2_-Au@Cu_2_O, photoactivity enhancements were observed at both UV and visible regions, showing a maximal AQY of 8.42 % at 300 nm and a peaked AQY of 2.74 % at 650 nm. Under UV irradiation, the photoexcited electrons and holes in TiO_2_ were spatially separated. The effective charge separation was accountable for the enlarged photoactivity in the UV region. On the other hand, the enlarged photoactivity in the visible range was ascribed to the plasmonic effect of Au and bandgap excitation from Cu_2_O. For TiO_2_-Au@Cu_7_S_4_, remarkable photoactivities of hydrogen production across UV and visible to near-infrared regions were observed. Resulting from Z-scheme charge transfer, the photoactivity under UV irradiation was largely enhanced, showing a noticeable AQY of 10.51 % at 300 nm. Remarkably, the Au@Cu_7_S_4_ decoration on the surface enabled TiO_2_ to absorb light across visible to near-infrared ranges through combined effects of LSPR from Au and Cu_7_S_4_, as well as the bandgap excitation of Cu_7_S_4_. The AQY can achieve 4.38, 4.17, and 3.66 % under 450, 800, and 1800 nm irradiation, respectively. It is noteworthy that the photoactivities of hydrogen production of current TiO_2_-Au@Cu_7_S_4_ were comparable and partly superior to the most advanced TiO_2_-based photocatalysts reported to date. To highlight this feature, we summarized the performance comparison among TiO_2_-based photocatalysts across UV, visible, and near-infrared regions in Tables S2–S4. Among the UV- and visible-responsive TiO_2_-based photocatalysts, the performance of current TiO_2_-Au@Cu_7_S_4_ was relatively ordinary. The best AQY achieved ever reported at UV and visible region was 38.6 % at 300 nm from PbS/(Pt-TiO_2_)^64^ and 45.6 % at 420 nm from CdS/TiO_2_@Ti_3_C_2_,^65^ substantially higher than the AQYs of the current TiO_2_-Au@Cu_7_S_4_. However, it should be emphasized that these seemingly highly efficient TiO_2_-based photocatalysts exhibited minimal responsiveness to near-infrared irradiation, a vast source of energy from natural sunlight that remains untapped. As noticed in Figure 7(b), the photoactivity of the current TiO_2_-Au@Cu_7_S_4_ can be enhanced across the whole near-infrared range by virtue of Cu_7_S_4_ showing LSPR. As Table S4 summarizes, among the near-infrared-responsive TiO_2_-based photocatalysts ever reported, TiO_2_-Au@Cu_7_S_4_ exhibited the highest AQY of 4.17 % at 800 nm and 3.66 % at 1800 nm. This characteristic is crucial for achieving full-spectrum-driven solar hydrogen production, as only a limited number of photocatalysts currently available can effectively respond to near-infrared irradiation.

It could be argued that surface characteristics, including specific surface area, porosity, and water adsorption, might affect the photocatalytic performance. To address this point, nitrogen adsorption–desorption isotherms were measured for two representative samples to evaluate specific surface areas and porosities. Table S5 shows that neither the surface area nor the pore diameter exhibited a clear correlation with photocatalytic efficiency. It is important to note that the pore diameters derived from nitrogen adsorption–desorption isotherm measurements may not accurately reflect the intrinsic porosity of the sampled materials. The measured average pore diameters were 40.11 nm for TiO_2_-Au@Cu_2_O and 24.52 nm for TiO_2_-Au@Cu_7_S_4_. These relatively large pore sizes likely resulted from the interstitial voids among the assembled nanowires. Nevertheless, the lack of a direct correlation with photocatalytic efficiency indicated that surface area and porosity did not have a predominant influence on the overall photocatalytic activity. On the other hand, to evaluate the possible influence of water adsorption on surface, the surface wettability properties for two representative samples were examined by assessing their contact angles. As shown in Figure S10, TiO_2_-Au@Cu_2_O exhibited a relatively large contact angle of 68.88°, indicating a hydrophobic surface. This hydrophobicity may be attributed to the predominant exposure of Cu_2_O (111) facets, as evidenced by the prominent Cu_2_O (111) diffraction peak at 36.50° in Figure 1(f).^66^ In contrast, TiO_2_-Au@Cu_7_S_4_ displayed a much smaller contact angle of 9.00°, reflecting its hydrophilic nature. Hydrophilic surfaces are known to adsorb water molecules more effectively, thereby facilitating the hydrolysis step during hydrogen production by enhancing the interaction between water and the catalyst surface. This improved water accessibility can promote hydrogen production. These wettability characteristics, as shown in Figure S10, help explain the superior hydrogen production performance of TiO_2_-Au@Cu_7_S_4_.

The shell thickness of Cu_7_S_4_ was likely crucial in modulating light absorption, charge transfer, and molecular diffusion, thereby influencing the overall photocatalytic efficiency of TiO_2_-Au@Cu_7_S_4_. In the synthesis of TiO_2_-Au@Cu_7_S_4_, Cu_7_S_4_ shell thickness can be easily adjusted by modulating Cu_2_O shell thickness of the parent Au@Cu_2_O.^67^ Shell thickness is a critical design parameter in yolk@shell structured photocatalysts as it directly influences light absorption, charge transfer dynamics, and molecular diffusion kinetics, all of which significantly impact photocatalytic performance. An optimal shell thickness is typically required: a shell that is too thin may result in insufficient photon harvesting, whereas an overly thick shell can hinder interfacial charge transfer and reduce the efficiency of molecular diffusion. The importance of this parameter has been well documented in the literature. For instance, Chen et al. showed that tuning the shell thickness of CdS in yolk@shell Au@CdS nanostructures significantly affected molecular diffusion kinetics across the shell, highlighting the presence of an optimal thickness for maximizing photocatalytic activity.^67^ Lee et al. investigated photoexcitation distances in yolk@shell Au@void@TiO_2_ structures and found that the penetration depth of light imposed a characteristic limit on the TiO_2_ shell thickness.^68^ While the current study focused primarily on constructing full-spectrum-responsive photocatalysts for solar hydrogen production, a systematic investigation on Cu_7_S_4_ shell thickness would provide valuable insight into further performance optimization.

Along with intrinsic activity, long-term stability is a key determinant in evaluating the practical feasibility of a photocatalyst. TiO_2_-Au@Cu_7_S_4_ was continuously used for 34 consecutive hours in solar hydrogen production to examine its long-term stability. As illustrated in Figure 7(c), the hydrogen production steadily and linearly increased with the duration of irradiation, revealing its high photostability during the long-term operation. The microstructural robustness was studied with XRD. Figure 7(d) shows that TiO_2_-Au@Cu_7_S_4_ exhibited minimal variation in the crystallographic structure following prolonged operation. If examined closely, a perceptible decrease in XRD peak intensity can be seen at 2θ = 14.19° and 15.20°. These two peaks resulted from the (110) and (002) facets of monoclinic TiO_2_. This suggested that these crystal planes might experience partial degradation during the reaction process. Nevertheless, microstructural characterizations of the used sample revealed that TiO_2_-Au@Cu_7_S_4_ retained good structural stability after the reaction. As shown in Figure S11, SEM, TEM, high-resolution TEM, SAED, and EDS analytical results confirmed that the overall morphology and elemental distribution of the used TiO_2_-Au@Cu_7_S_4_ remained well preserved. The high photostability and microstructural integrity spotlight the practice and utility of current TiO_2_-Au@Cu_7_S_4_ toward advanced photocatalytic applications.

Thermal stability is another key factor in assessing the practical viability of photocatalysts. To assess this characteristic, thermogravimetric analysis (TGA) and temperature-dependent XRD measurements were performed on TiO_2_-Au@Cu_7_S_4_. As shown in Figure S12­(a), three regions of weight change were observed. The weight decrease (1.170 %) observed from room temperature to 210 °C was believed to result from desorption of physisorbed water. A minor weight decrease (0.438 %) in the 210–480 °C range likely corresponded to the removal of chemisorbed water and residual nonhydrolyzed isopropoxide groups. No significant weight loss was observed beyond this point up to 1000 °C. The overall weight loss of only 1.608 % confirmed the robust thermal resilience for TiO_2_-Au@Cu_7_S_4_. In addition, temperature-dependent XRD analysis was performed to assess the structural stability. The as-synthesized TiO_2_-Au@Cu_7_S_4_ was first measured at room temperature; afterward, it was heated at 100 °C for 5 min followed by another XRD measurement. This procedure was repeated in 100 °C increments up to 600 °C. As illustrated in Figure S12­(b), the XRD patterns at all elevated temperatures remained consistent with those measured at room temperature. These results confirmed that TiO_2_-Au@Cu_7_S_4_ maintained its structural integrity even under elevated temperatures, further demonstrating its good thermal stability.

To further highlight the superior feature of monoclinic-phase TiO_2_ in photocatalytic hydrogen production, a comparative analysis of TiO_2_ photocatalysts with different crystalline phases (anatase, rutile, and monoclinic) has been summarized in Table S7. Based on this literature survey, it is acknowledged that anatase-phase TiO_2_ often exhibits high photocatalytic hydrogen production activity, attributed to its favorable band edge alignment and reduced charge recombination rate. However, it has also been noted that monoclinic-phase TiO_2_ can outperform anatase TiO_2_ under specific conditions. For instance, in Li’s study,^69^ Ti^3+^ self-doped monoclinic TiO_2_ achieved a hydrogen yield of 9372.9 μmol h^–1^ g^–1^ in 18.5 % methanol solution, whereas anatase TiO_2_ achieved a hydrogen yield of 4029.4 μmol h^–1^ g^–1^ under identical conditions. Similarly, in a 20 % methanol electrolyte, monoclinic TiO_2_ attained a hydrogen yield (40.8 μmol h^–1^ g^–1^) that was much better than anatase TiO_2_ (5.6 μmol h^–1^ g^–1^).^70^ These findings suggested that despite the general consensus on anatase superiority, monoclinic TiO_2_ can exhibit competitive or even superior performance when tailored with optimized heterostructures.

Drawing from the experimental results, a reasonable mechanism was suggested to elucidate the enhanced solar hydrogen production efficiency of TiO_2_-Au@Cu_7_S_4_. As illustrated in Figure 8, under bandgap excitation by UV and visible lights, the photoexcited carriers were produced in TiO_2_ and Cu_7_S_4_. Under the Z-scheme charge transfer scenario, the photoexcited electrons were accumulated at Cu_7_S_4_, spatially separating from the photogenerated holes concentrated at TiO_2_. The significant charge separation contributed to the marked improvement in photoactivity within the 300 to 600 nm spectral range. Under LSPR excitation by visible and near-infrared lights, the hot charge carriers generated at Au and Cu_7_S_4_ as well as the magnified localized electric field contributed to the additional photoactivity enhancement beyond 600 nm. By decorating TiO_2_ with Au@Cu_7_S_4_, the current TiO_2_-Au@Cu_7_S_4_ can achieve noticeable photoactivity of hydrogen production across UV, visible, and near-infrared regions, providing a new photocatalyst paradigm for the realization of solar hydrogen production.

**Figure 8:**
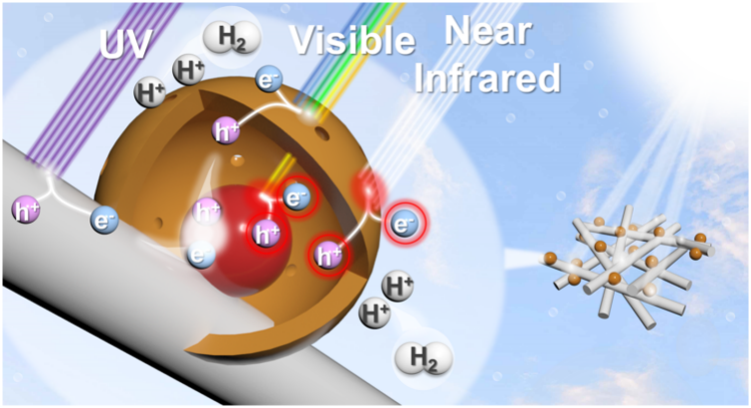
Proposed charge transfer mechanisms for TiO_2_-Au@Cu_7_S_4_ under a bandgap and plasmonic excitations toward solar hydrogen production.

## Conclusions

To summarize, the TiO_2_-Au@Cu_7_S_4_ heterostructure nanowires exhibit notable efficiency as photocatalysts for solar-driven hydrogen production by effectively utilizing the entire solar spectrum. By coupling the Z-scheme mechanism, bandgap absorption of Cu_7_S_4_, the LSPR effects of both Au and Cu_7_S_4_, and the surficial hydrophilicity, the present TiO_2_-Au@Cu_7_S_4_ nanostructures demonstrated a high efficiency in solar hydrogen production across a wide spectral region, achieving notable AQYs of 10.51 % at 300 nm, 4.38 % at 450 nm, 4.17 % at 800 nm, and 3.66 % at 1800 nm. The near-infrared photoactivity of TiO_2_-Au@Cu_7_S_4_ was remarkable, surpassing the most advanced near-infrared-responsive TiO_2_-based photocatalysts reported to date. The present study delivers a new type of full-spectrum-responsive photocatalyst paradigm enabling efficient hydrogen production from the untapped near-infrared energy. The present study offers a viable strategy of broadening the photoresponse range for the well-established but dependable TiO_2_-based photocatalysts, paving the way for their utility in solar-driven photocatalysis.

## Supplementary Material



## Notes

Experimental details; calculations of loading amounts for photocatalysts; TEM-EDS mapping profiles; Tauc plots; XPS spectra; UPS spectra; HAADF; HRTEM images; SAED patterns; SEM images; hydrogen production data; band alignment; contact angle measurements; TGA profiles; XRD patterns; and global activity comparison (PDF)
