# Supplementary material for: Full-Spectrum-Responsive Au@Cu7S4‑Decorated Monoclinic TiO2 Nanowires for Solar Hydrogen Production
Source: ACS Appl Mater Interfaces. 2025 Jun 17;17(28):40340–52. doi: 10.1021/acsami.5c03462 (PMC12278245; doi:10.1021/acsami.5c03462)
Supplement: Supplementary file 1 [file am5c03462_si_001.pdf]

Supporting Information

Full-Spectrum-Responsive Au@Cu_7S_4-Decorated Monoclinic TiO_2 Nanowires for Solar Hydrogen Production

Yu-Ting Wang,[a] *Hsuan-Hung Kuo,*[a] *Chun-Yi Chen,*[b]* *Tso-Fu Mark Chang,*[b]* *Masato Sone,*[b] *Yung-Jung Hsu*[a, b, c]*

[a] Department of Materials Science and Engineering, National Yang Ming Chiao Tung University, Hsinchu 300093, Taiwan

[b] Institute of Integrated Research, Institute of Science Tokyo, Kanagawa 226-8503, Japan

[c] Center for Emergent Functional Matter Science, National Yang Ming Chiao Tung University, Hsinchu 300093, Taiwan

*Email: chen.c.ac@m.titech.ac.jp; chang.m.aa@m.titech.ac.jp; yhsu@cc.nctu.edu.tw

Experimental Section

Chemicals

All chemicals used in this study were of analytical grade and purchased from Sigma-Aldrich. These included commercial TiO_2 powders (Degussa P-25), absolute ethanol (99.5 %), poly(N-vinylpyrrolidone) powder (denoted as PVP, Mw = 10,000 Da, 99 %), copper (II) sulfate hydrate ($CuSO_4 \cdot 5H_2O$, 99-102 %), copper (II) chloride ($CuCl_2$, 97 %), sodium hydroxide (NaOH, 100 %), L-ascorbic acid ($C_6H_8O_6$, denoted as LAA, 100 %), sodium citrate dehydrate ($C_6H_5Na_3O_7 \cdot 2H_2O$, 99.0 %), sodium sulfide nonahydrate (Na_2S, 98+ %), sodium sulfite anhydrous (Na_2SO_3, 98+ %), sodium sulfate anhydrous (Na_2SO_4, 99+ %), methanol (CH_3OH, 99.8 %), ethanol (C_2H_5OH, 99.5 %), triethanolamine ($C_6H_{15}NO_3$, denoted as TEOA, 99.00 %), lead nitrate ($Pb(NO_3)_2$, 99.8 %), and silver nitrate ($AgNO_3$, 99.8 %). All aqueous solutions were prepared using high-purity deionized (DI) water as the solvent.

Synthesis of Monoclinic TiO_2 Nanowires

Monoclinic TiO_2 nanowires were synthesized using Degussa P-25 powders as the starting material. In a typical procedure, 1.0 g of P-25 was dispersed in 10 mL of absolute ethanol, followed by the addition of 10.0 mL of 10 M NaOH aqueous solution under vigorous stirring. The resulting suspension was transferred to a Teflon-lined stainless steel autoclave and subjected to a hydrothermal treatment at 200 °C for 24 h. After cooling to room temperature, the precipitates were collected and washed repeatedly with DI water and hydrochloric acid (HCl) solution (pH = 1) until the filtrate reached neutral pH (pH = 7). The final product was dried and subsequently annealed in air at 700 °C for 4 h to obtain TiO_2 nanowires in monoclinic phase.[1]

Decoration of Au Particles

Au particles with an approximate size of 15 nm were initially synthesized through a standard citrate reduction method.[2] The citrate-stabilized Au colloidal solution (100.0 mL, 0.25 mM) was mixed with 10.0 mL of 0.01 M PVP solution to facilitate surface ligand exchange. The resulting PVP-stabilized Au particles (0.025 mmol) were then isolated by centrifugation at 14,000 rpm, rinsed with DI water, and re-dispersed into DI water (100.0 mL) to form a PVP-stabilized Au particle suspension. To functionalize TiO_2 nanowires with Au particles, 10.0 mg of TiO_2 nanowires were distributed in 30.0 mL of DI water, followed by the addition of 3.0 mL of the PVP-stabilized Au particle suspension. After stirring for 30 min, the Au-decorated TiO_2 (denoted as TiO_2-Au) was separated by centrifugation at 9,000 rpm and washed with DI water. The Au content of TiO_2-Au was determined to be

0.894 wt% by inductively coupled plasma mass spectrometry (ICP-MS). For comparison purpose, three different volumes of PVP-stabilized Au particle suspension were employed (1.5 mL, 3.0 mL, and 4.5 mL) to produce TiO_2-Au with increasing Au loading amounts. The resultant products were denoted as TiO_2-Au-1.5, TiO_2-Au-3.0, and TiO_2-Au-4.5, respectively. Among the series, TiO_2-Au-3.0 exhibited the highest photocatalytic activity. Therefore, TiO_2-Au-3.0 was further employed for the deposition of Cu_2O and Cu_7S_4.

Deposition of Cu_2O and Cu_7S_4

Cu_2O was deposited onto the Au surface of TiO_2-Au via a chemical reduction method.[3] Initially, 10.0 mg of TiO_2-Au was dispersed in 16 mL of DI water, followed by the addition of 2.0 mL of 0.01 M $CuSO_4$ solution. Subsequently, 1.5 mL of 0.1 M NaOH aqueous solution was introduced, along with a rapid injection of 0.5 mL of 0.1 M LAA solution. After vigorous stirring at 35 °C for 10 min, the reaction solution turned dark green, signifying the deposition of Cu_2O on the Au surface of TiO_2-Au. The resulting product, Au@Cu_2O-decorated TiO_2 (denoted as TiO_2-Au@Cu_2O), was isolated by centrifugation at 9,000 rpm and washed with DI water. According to the ICP-MS analysis, the Au and Cu contents of TiO_2-Au@Cu_2O were respectively determined to be 0.760 and 9.134 wt%. To convert Cu_2O into Cu_7S_4, 0.4 mL of 0.1 M Na_2S aqueous solution was added to the TiO_2-Au@Cu_2O suspension. The mixture was stirred at 35 °C for 15 min, during which the reaction solution turned brown, indicating the conversion of Cu_2O to Cu_7S_4. The final product, Au@Cu_7S_4-decorated TiO_2 (denoted as TiO_2-Au@Cu_7S_4), was collected by centrifugation at 9,000 rpm, washed with both DI water and HCl solution (pH = 1), and dried under vacuum for further use. According to the ICP-MS analysis, the Au, Cu, and S contents of TiO_2-Au@Cu_7S_4 were respectively determined to be 0.779, 9.326, and 2.804 wt%. For comparison, plain Au@Cu_2O and plain Au@Cu_7S_4 nanocrystals were synthesized following the same procedures, excluding the TiO_2 nanowires. Pure Cu_2O and pure Cu_7S_4 were also prepared using identical methods but without the addition of Au particles. These four samples served as a counterpart to the corresponding heterostructure nanowires.

Photocatalytic Hydrogen Production

For the photocatalytic hydrogen production experiments, a solar simulator (Newport, LCS-100, 94011A), equipped with a xenon lamp and an AM 1.5 G filter (100 mW/cm^2), was used as the irradiation source. A specified amount of sample powder was dispersed in 40.0 mL of 0.5 M Na_2SO_3 aqueous solution within a quartz reactor (4.0 cm in length × 4.0 cm in width × 7.0 cm in height). Four TiO_2-based

samples were employed as photocatalysts and compared, including pristine TiO_2, TiO_2-Au, TiO_2-Au@Cu_2O, and TiO_2-Au@Cu_7S_4. For photocatalytic experiments, the amount of the TiO_2 component for the four TiO_2-based samples was fixed in order to explore the influence of the decorated Au, Au@Cu_2O and Au@Cu_7S_4 on the photocatalytic efficiency of TiO_2. To give a fixed TiO_2 amount of 5.00 mg, the loading weight of pristine TiO_2, TiO_2-Au, TiO_2-Au@Cu_2O, and TiO_2-Au@Cu_7S_4 was determined to be 5.00 mg, 5.03 mg, 5.75 mg, and 5.85 mg, respectively. The detailed calculations for the determination of the loading amounts were specified in the next section. Besides the above four TO_2-nased samples, four additional physical mixture samples were also employed and compared. Here, the four physical mixtures were prepared by simply mixing pristine TiO_2 with Au, Au@Cu_2O, Au@Cu_7S_4, or pure Cu_7S_4. The resultant samples were noted as TiO_2+Au, TiO_2+Au@Cu_2O, TiO_2+Au@Cu_7S_4, and TiO_2+Cu_7S_4, respectively. For the four physical mixtures, the loading amount was equivalent to the corresponding heterostructure nanowire samples. For example, for TiO_2+Au@Cu_7S_4, 5.00 mg of TiO_2 was mixed with 0.85 mg of Au@Cu_7S_4, giving a total loading amount of 5.85 mg for conducting photocatalytic reaction. Prior to irradiation, the reaction solution was purged with argon gas for 1 h to eliminate dissolved gas species. The reactor was then sealed and subjected to irradiation for the hydrogen production reaction. At one-hour intervals, 1.0 mL of gas from the reactor headspace was withdrawn using a syringe. The collected gas was analyzed via gas chromatography (Bruker SCION, 463-MS) to measure the amount of hydrogen produced. The recycling tests of the samples were conducted by consecutively performing photocatalytic hydrogen production reactions for 34 h. The apparent quantum yield (AQY) of hydrogen production was calculated using equation (S1):

$$\text{AQY}\ (\%) = \frac{\text{number of reacted electrons}}{\text{number of incident photons}} \times 100\ \% = \frac{2\times\text{number of hydrogen molcules}}{\text{number of incident photons}} \times 100\ \% \quad \text{(S1)}$$

Site-Selective Photo-deposition of Ag and PbO_2

The site-specific photodeposition of Ag and PbO_2 onto distinct regions of TiO_2-Au@Cu_7S_4 was conducted following the reported procedures.[4] To selectively deposit Ag on the Cu_7S_4 surface, 1.0 mg of TiO_2-Au@Cu_7S_4 was dispersed in 40 mL of DI water, followed by the addition of 300 μL of 0.2 mM $AgNO_3$ solution and 300 μL of 4 mM sodium citrate solution as the Ag source and stabilizing agent, respectively. The resulting mixture was then irradiated under AM 1.5 G illumination (100 mW/cm^2) for 1 h. To selectively deposit PbO_2 on the TiO_2 surface, 1.0 mg of TiO_2-Au@Cu_7S_4 was dispersed in 40 mL of DI water, followed by the addition of 300

μL of $Pb(NO_3)_2$ solution (0.01 M). The same procedures were also used to perform site-selective photo-deposition experiments on TiO_2-Au@Cu_2O.

Characterizations

The morphology and dimensions of the samples were characterized using a field-emission scanning electron microscope (SEM, Hitachi SU8010). Atomic-scale structural analysis was conducted using a scanning transmission electron microscope (STEM, JEOL JEM-ARM200FTH) equipped with a spherical aberration corrector and a high-angle annular dark-field (HAADF) detector. Elemental distribution was examined via energy-dispersive X-ray spectroscopy (EDS) integrated into the STEM system. Crystallographic information was obtained from X-ray diffraction (XRD) patterns collected using a Bruker D2 Phaser diffractometer. UV–visible–near infrared diffuse reflectance spectra (DRS) were recorded on a Hitachi U-3900H spectrophotometer equipped with an integrating sphere. Steady-state photoluminescence (PL) spectra were recorded on a Hitachi F-4500 spectrofluorometer with an excitation wavelength of 325 nm. Time-resolved PL measurements were recorded in a customized single-photon counting system, employing a sub-nanosecond pulsed diode laser (λ = 375 nm, PicoQuant PLD 375) with an instrument response function (IRF) of approximately 50 ps full width at half maximum. X-ray photoelectron spectroscopy (XPS) measurements were carried out on a Thermo Fisher Scientific ESCALAB Xi+ spectrometer using Al Kα radiation, and all binding energies were calibrated against the C 1s peak at 284.8 eV. Ultraviolet photoelectron spectroscopy (UPS) was performed on a ULVAC-PHI PHI 5000 Versaprobe II system with He I excitation ($h\nu$ = 21.22 eV). Thermal stability was assessed by thermogravimetric analysis (TGA) using a TA Instruments SDT 650 under a nitrogen atmosphere. TGA analysis was conducted on TiO_2-Au@Cu_7S_4 using 1.76 mg of dried powder. The sample was heated from room temperature to 1000 °C at a rate of 10 °C min^{-1} under nitrogen atmosphere. The specific surface area and pore size distribution were determined using nitrogen adsorption–desorption isotherms at 77 K with a Micromeritics ASAP 2020 analyzer, applying the Brunauer–Emmett–Teller (BET) and Barrett–Joyner–Halenda (BJH) models, respectively. Elemental compositions were analyzed with ICP-MS (Thermo Fisher Scientific iCAP TQ) by acid digestion of the samples.

Calculations of loading amounts for photocatalysts

Four TiO_2-based samples were employed as photocatalysts and compared, including pristine TiO_2, TiO_2-Au, TiO_2-Au@Cu_2O, and TiO_2-Au@Cu_7S_4. For photocatalytic experiments, the amount of the TiO_2 component for the four samples was fixed in order to explore the influence of the decorated Au, Au@Cu_2O, and Au@Cu_7S_4 on the photocatalytic efficiency of TiO_2. The detailed calculations of the loading amounts were specified by equations S(2), S(3) and S(4) as follows.

(1) TiO_2-Au: The synthesis mainly used 10.0 mg TiO_2 and 3.0 mL PVP-stabilized Au. Here, 3.0 mL PVP-stabilized Au was sampled from 100.0 mL PVP-stabilized Au which originally contained 0.025 mmol Au. The weight of the decorated Au was estimated to be 0.059 mg. The as-synthesized TiO_2-Au thus comprised 10.0 mg TiO_2 and 0.059 mg Au. To give a fixed TiO_2 amount of 5.0 mg, the loading weight of TiO_2-Au was determined to be (10.0 + 0.059) ÷ 2 = 5.030 mg. The quantity of TiO_2-Au used in photocatalytic hydrogen production was obtained by rounding the theoretically calculated value to the second decimal place, i.e. 5.03 mg, in accordance with the precision limits of the analytical balance.

$$Weight\ of\ Au = n_{Au} \times MW_{Au} = \left(\frac{3}{100} \times 0.025 \times 10^{-3}\right) \times 79 = 0.000059\ g = 0.059 mg \quad S(2)$$

(2) TiO_2-Au@Cu_2O: The synthesis mainly used 10.0 mg TiO_2, 3.0 mL PVP-stabilized Au, and 2.0 mL $CuSO_4$ (0.01 M). The weight of the deposited Cu_2O was estimated to be 1.431 mg. The as-synthesized TiO_2-Au@Cu_2O thus comprised 10.0 mg TiO_2, 0.059 mg Au, and 1.431 mg Cu_2O. To give a fixed TiO_2 amount of 5.0 mg, the loading weight of TiO_2-Au@Cu_2O was determined to be (10.0 + 0.059 + 1.431) ÷ 2 = 5.745 mg. The quantity of TiO_2-Au@Cu_2O used in photocatalytic hydrogen production was obtained by rounding the theoretically calculated value to the second decimal place, i.e. 5.75 mg, in accordance with the precision limits of the analytical balance.

$$Weight\ of\ Cu_2O = n_{Cu2O} \times MW_{Cu2O} = \left(2 \times 0.01 \times 10^{-3} \times \frac{1}{2}\right) \times \left((63.546 \times 2) + 16\right) = 0.001431\ g = 1.431\ mg \quad S(3)$$

(3) TiO_2-Au@Cu_7S_4: The synthesis mainly used 10.0 mg TiO_2, 3.0 mL PVP-stabilized Au, and 2.0 mL $CuSO_4$ (0.01 M). The weight of the deposited Cu_7S_4 was estimated to be 1.637 mg. The as-synthesized TiO_2-Au@Cu_2O thus comprised 10.0 mg TiO_2, 0.059 mg Au, and 1.637 mg Cu_7S_4. To give a fixed TiO_2 amount of 5.0 mg, the loading weight of TiO_2-Au@Cu_7S_4 was determined to be (10.0 + 0.059 + 1.637) ÷ 2 =

5.848 mg. The quantity of TiO_2-Au@Cu_7S_4 used in photocatalytic hydrogen production was obtained by rounding the theoretically calculated value to the second decimal place, i.e. 5.85 mg, in accordance with the precision limits of the analytical balance.

$$Weight\ of\ Cu_7S_4 = n_{Cu7S4} \times MW_{Cu7S4} = \left(2 \times 0.01 \times 10^{-3} \times \frac{1}{7}\right) \times \left((63.546 \times 7) + (32 \times 4)\right) = 0.001637\ g = 1.637\ mg \quad S(4)$$

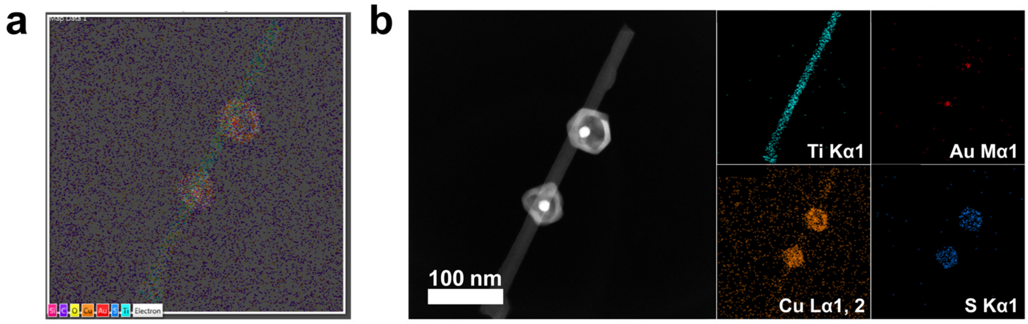

Figure S1. (a) Overlapped TEM-EDS mapping profiles and (b) corresponding HAADF image and EDS mapping data for TiO_2-Au@Cu_7S_4.

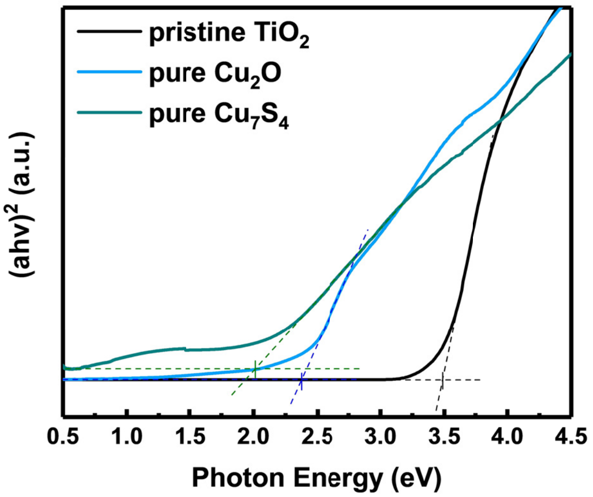

Figure S2. Corresponding Tauc plots for determining the bandgap for TiO_2, Cu_2O, and Cu_7S_4.

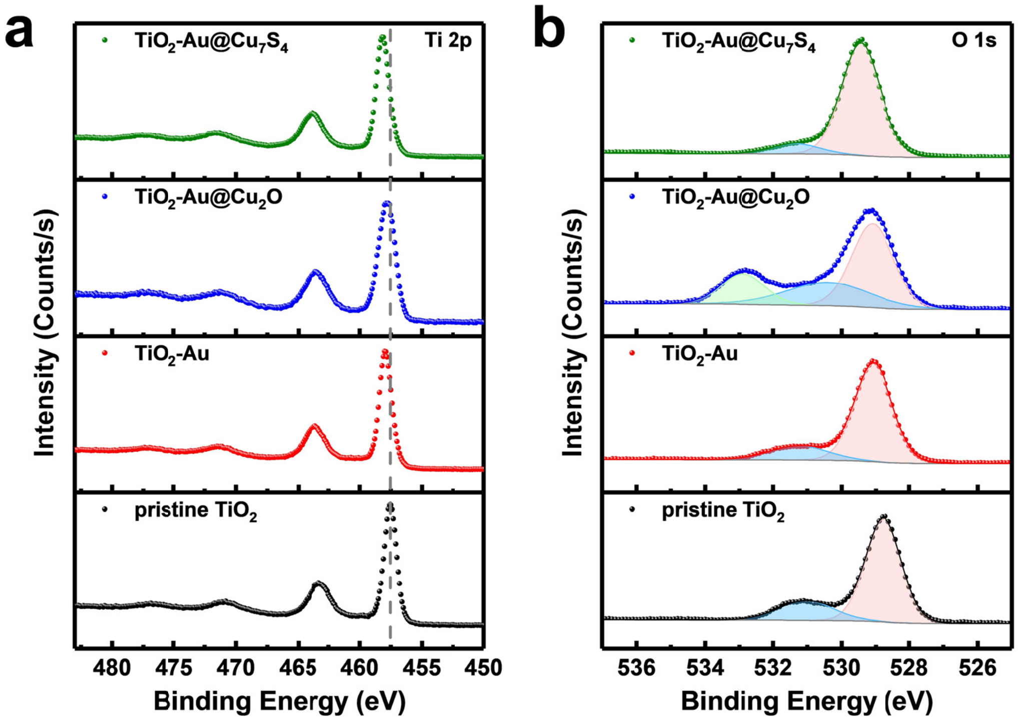

Figure S3. XPS spectra for four TiO_2-based nanowire samples in (a) Ti 2p and (b) O 1s core level.

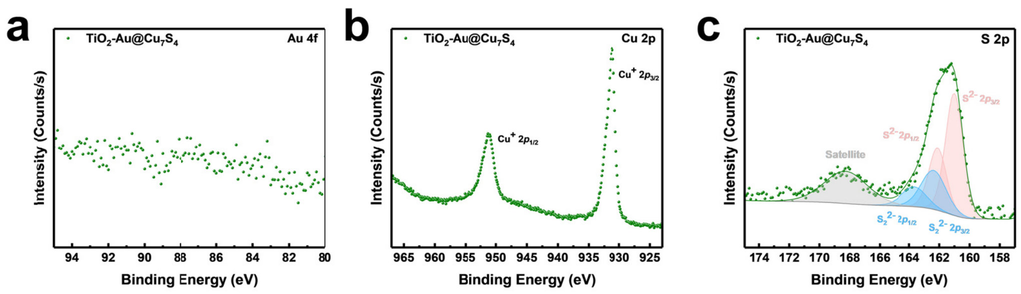

Figure S4. XPS spectra of (a) Au 4f, (b) Cu 2p, and (c) S 2p for TiO_2-Au@Cu_7S_4.

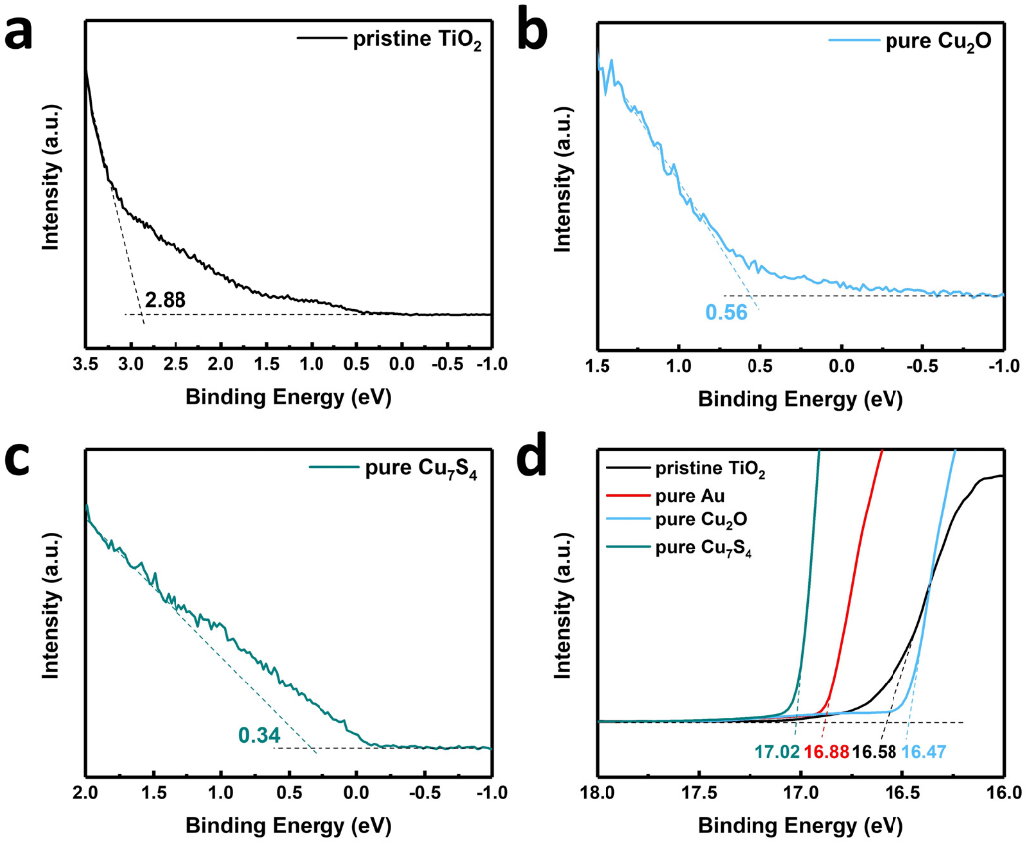

Figure S5. UPS spectra recorded in the valence band distribution region for (a) pristine TiO_2, (b) pure Cu_2O, and (c) pure Cu_7S_4. (d) UPS spectra recorded in the secondary-electron cut-off region for the four components.

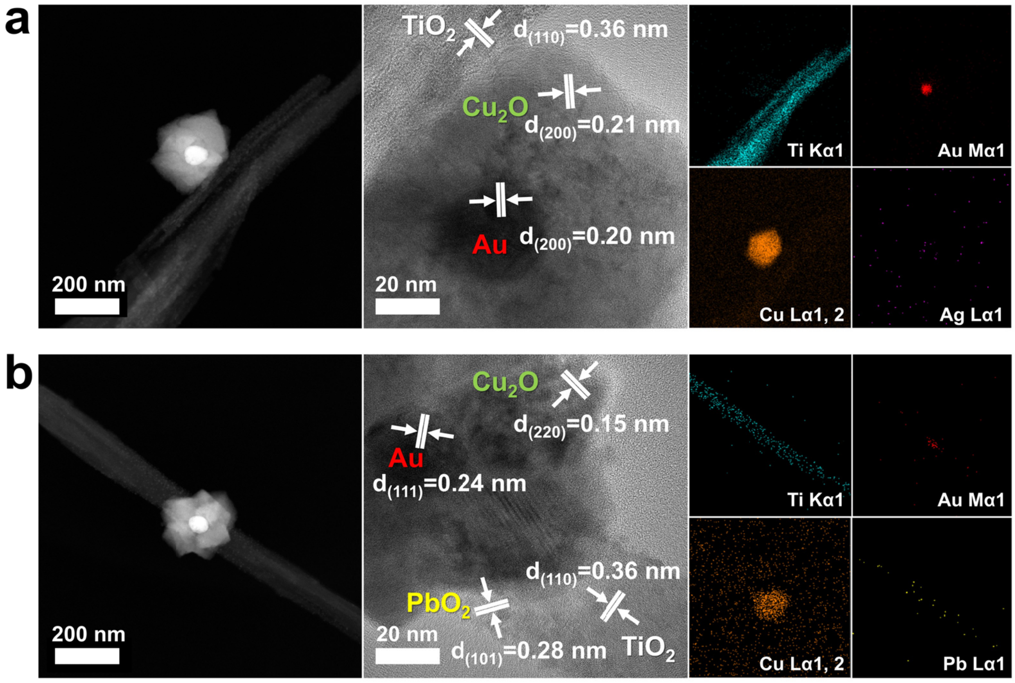

Figure S6. HAADF, HRTEM images and TEM-EDS mapping profiles for site-selective photodeposition of (a) Ag and (b) PbO_2 on TiO_2-Au@Cu_2O.

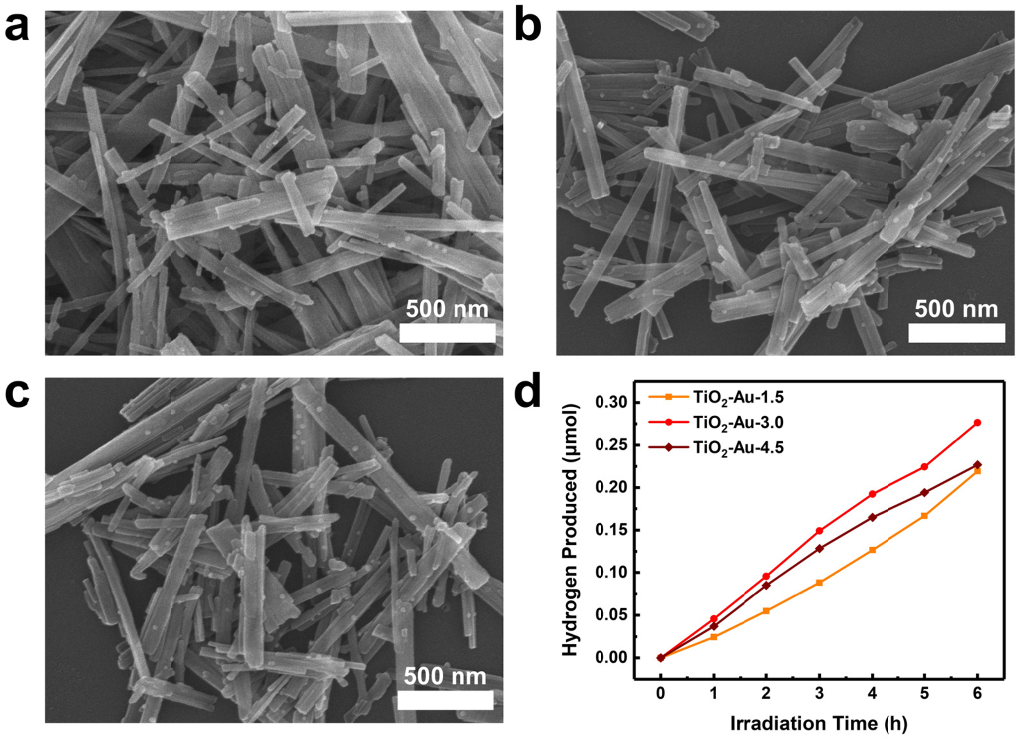

Figure S7. SEM images of (a) TiO_2-Au-1.5, (b) TiO_2-Au-3.0, (c) TiO_2-Au-4.5. (d) Comparison of hydrogen production activity among the three TiO_2-Au.

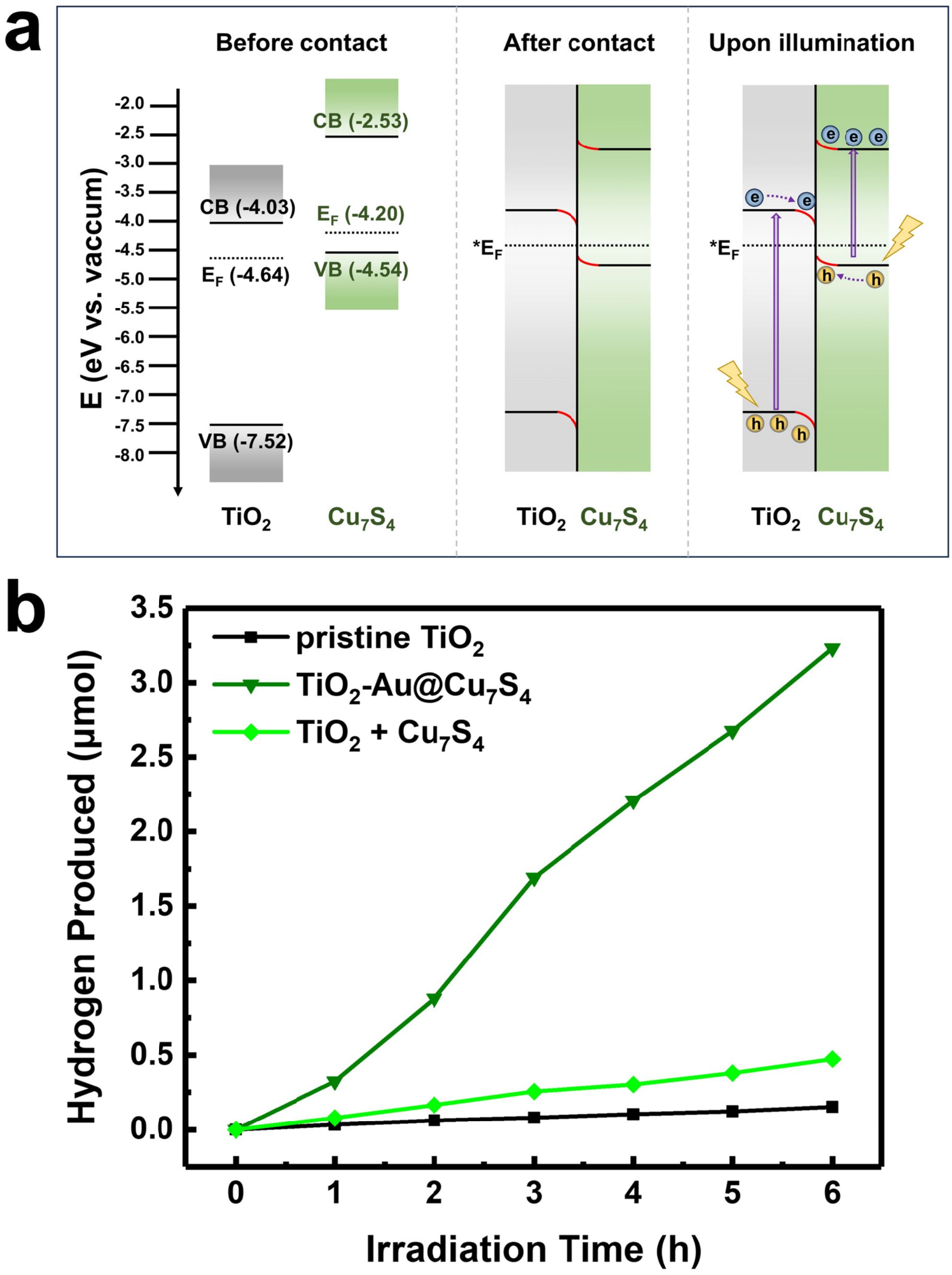

Figure S8. (a) Proposed band alignment and charge transfer mechanisms for TiO_2+Cu_7S_4. (b) Comparison of hydrogen production activity among pristine TiO_2, TiO_2-Au@Cu_7S_4, and TiO_2+Cu_7S_4.

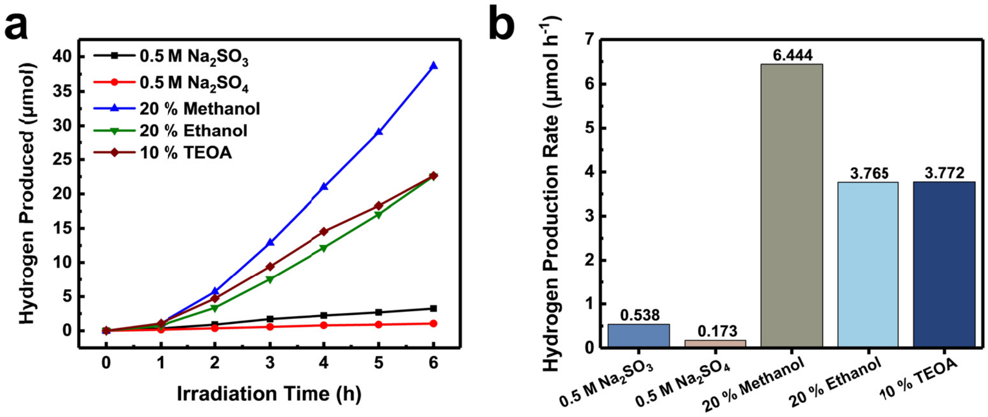

Figure S9. Comparison of hydrogen production activity among five relevant sacrificial agents over TiO_2-Au@Cu_7S_4 in (a) total amount of hydrogen produced and (b) hydrogen yield.

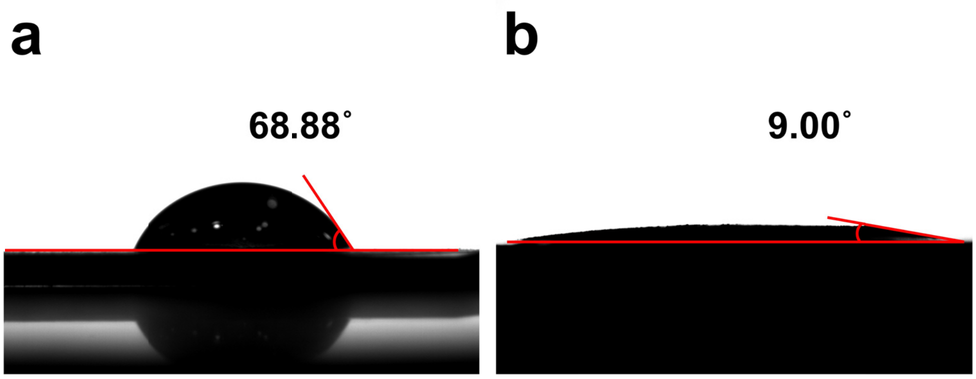

Figure S10. Images of a water droplet on the surface of (a) TiO_2-Au@Cu_2O and (b) TiO_2-Au@Cu_7S_4. The measured contact angles were marked for clarity.

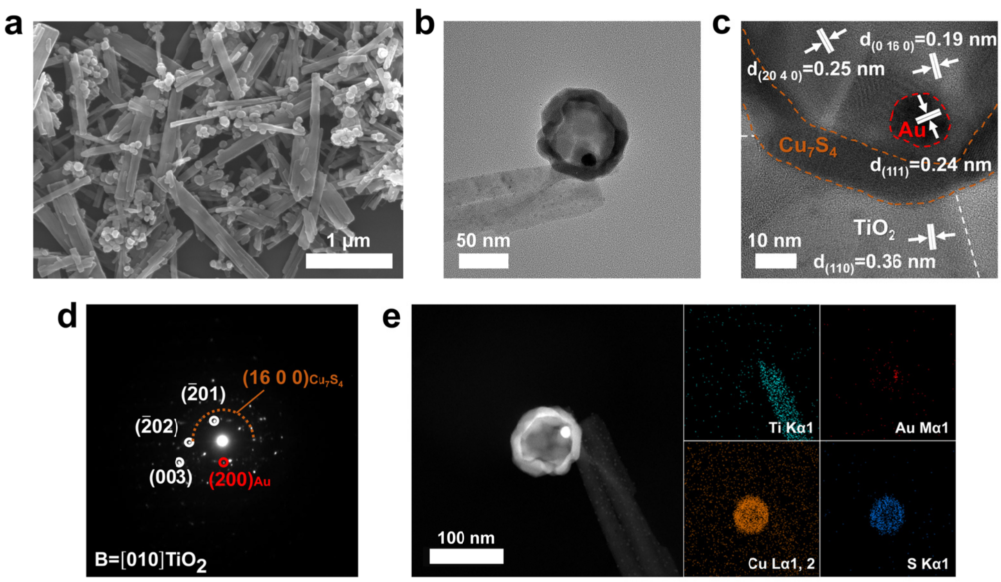

Figure S11. (a) SEM image, (b) TEM image, (c) high-resolution TEM image, (d) SAED pattern, and (e) HAADF image and EDS mapping data for TiO_2-Au@Cu_7S_4 after used in solar hydrogen production.

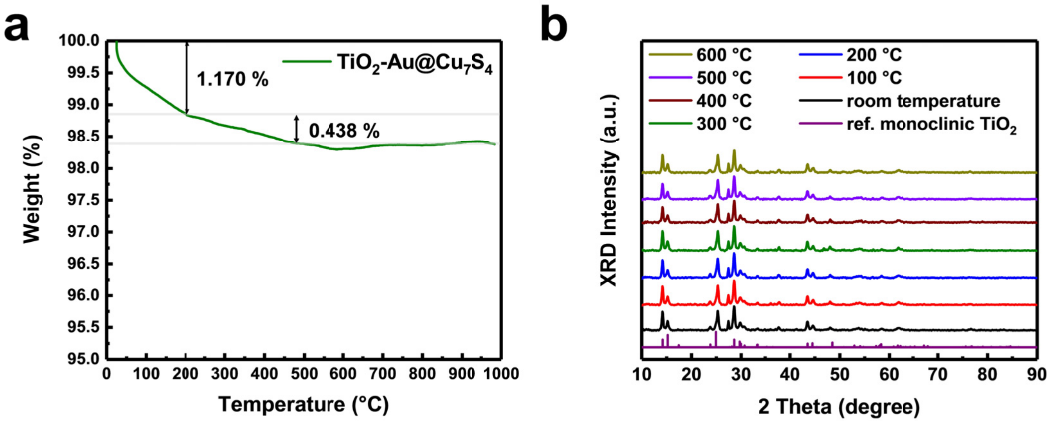

Figure S12. (a) TGA curve of TiO_2-Au@Cu_7S_4. (b) Temperature-dependent XRD patterns of TiO_2-Au@Cu_7S_4 after annealing at 100 ˚C, 200 °C, 300 °C, 400 °C, 500 °C, and 600 °C.

Table S1. Fitting results of time-resolved PL spectra for the four TiO_2-based samples.

Sample	A_1 (%)	τ_1 (ns)	A_2 (%)	τ_2 (ns)	$\langle\tau\rangle$ (ns)	χ^2	k_{ct} (s^{-1})
pristine TiO_2	38.1	5.33	61.9	1.22	4.22	1.01	
TiO_2-Au	37.6	5.19	62.4	1.17	4.10	1.02	7.20×10^6
TiO_2-Au@Cu_2O	37.5	4.89	62.5	1.16	3.83	1.02	2.42×10^7
TiO_2-Au@Cu_7S_4	33.1	4.54	66.9	1.02	3.44	1.02	5.34×10^7

Table S2. Performance comparison of photocatalytic H_2 production with the state-of-the-art TiO_2-based, UV-responsive photocatalysts reported in the literature in the recent three years.

Catalysts [co-catalyst]	Light Source	Electrolyte	Activity	Ref.
TiO_2-Au@Cu_7S_4 [none]	AM 1.5 G 100 mW/cm^2	Na_2SO_3 (0.5 M)	92.08 μmol h^{-1} g^{-1} AQY = 10.51 % (300 nm)	This work
[1]2-NiO/TiO_2 [none]	Hg lamp, 8W 1.213 mW/cm^2	Methanol (50 %)	233.67 μmol h^{-1} g^{-1} AQY = 2.9 % (254 nm)	(5)
[2]1.5Ag/TiO_2 [none]	UV light 4.40 mW/cm^2	Na_2S (0.1 N) and Na_2SO_3 (0.1 N)	23496 μmol h^{-1} g^{-1} AQY = 19 % (254 nm)	(6)
5.0 wt% PbS/(Pt-TiO_2) [1.0 wt% Pt]	Xenon lamp 100 mW/cm^2	Na_2S (0.35 M) and Na_2SO_3 (0.25 M)	16260 μmol h^{-1} g^{-1} AQY = 38.6 % (350nm)	(7)
[3]C-1.5/T-2 [none]	Xenon lamp 100 mW/cm^2	Methanol (30 %)	11000 μmol h^{-1} g^{-1} AQY = 15.1 % (365 nm)	(8)
Pd-TiO_2 [none]	Xenon lamp [5]N. A.	Methanol (25 %)	24590 μmol h^{-1} g^{-1} AQY = 23.4 % (365 nm)	(9)
[4]TiO_2-BNS-Pt [0.05wt% Pt]	Xe arc lamp 170 mW/cm^2	Methanol (50 %)	688.7 μmol h^{-1} g^{-1} AQY = 8.48 % (325 nm)	(10)

[1]2-NiO/TiO_2 stands for NiO-decorated TiO_2 prepared by adding TiO_2 to 0.2 M nickelnitrate solution.

[2]1.5Ag/TiO_2 stands for Ag-doped TiO_2 prepared by adding TiO_2 to $AgNO_3$ aqueous solution containing 1.5 g of Ag^+ per 100 g TiO_2.

[3]C-1.5/T-2 stands for Cu_2O-decorated TiO_2 prepared by adding ethylene glycol-derived TiO_2 (denoted as T-2) to 1.5 mM $Cu(CH_3COO)_2$ solution.

[4]TiO_2-BNS-Pt stands for bronze-phase TiO_2 nanosheets (TiO_2-BNS) loaded with 0.05 wt % Pt.

[5]N. A. means not available, i.e. the irradiation power was not provided.

Table S3. Performance comparison of photocatalytic H_2 production with the state-of-the-art TiO_2-based, visible-responsive photocatalysts reported in the literature in the recent three years.

Catalysts [co-catalyst]	Light Source	Electrolyte	Activity	Ref.
TiO_2-Au@Cu_7S_4 [none]	AM 1.5 G 100 mW/cm^2	Na_2SO_3 (0.5 M)	92.08 μmol h^{-1} g^{-1} AQY = 4.38 % (450 nm)	This work
[1]P25/Pt/1 [none]	Xenon lamp 100 mW/cm^2	Ascorbic acid (0.5 M)	[7]N. A. AQY = 3.80 % (420 nm)	(11)
[2]R-CQD/P2-2.5 [none]	Xenon lamp 158 mW/cm^2	Methanol (25 %)	2190 μmol h^{-1} g^{-1} AQY = 4.94 % (450 nm)	(12)
CdS/TiO_2@Ti_3C_2 [none]	Xenon lamp [6]N. A.	Triethanolamine (10 %)	623 μmol h^{-1} g^{-1} AQY = 45.6 % (420 nm)	(13)
5.0 wt% PbS/(Pt–TiO_2) [1.0 wt% Pt]	Xenon lamp 100 mW/cm^2	Na_2S (0.35 M) and Na_2SO_3 (0.25 M)	16260 μmol h^{-1} g^{-1} AQY = 8.35 % (450nm)	(7)
[3]15-ZCS/TiO_2 [none]	Xenon lamp [6]N. A.	Triethanolamine (10 %)	5580 μmol h^{-1} g^{-1} AQY = 11.5 % (420 nm)	(14)
[4]S5@Pt/TiO_2 [none]	Xenon lamp [6]N. A.	Ascorbic acid (10 %)	21500 μmol h^{-1} g^{-1} AQY = 1.33 % (420 nm)	(15)
[5]CuPc/TiO_2 [none]	Xenon lamp [6]N. A.	Methanol (40 %)	95 μmol h^{-1} g^{-1} AQY = 6.68 % (420 nm)	(16)

[1]P25/Pt/1 stands for Pt-loaded P-25 TiO_2 sensitized by benzofuran[b]-fused BODIPY with thienyl-cyanoacrylic acid (denoted as 1).

[2]R-CQD/P25–2.5 stands for P-25 TiO_2 decorated with 2.5 wt % red emissive carbon quantum dots (denoted as R-CQD).

[3]15-ZCS/TiO_2 stands for TiO_2 loaded with 15 % $ZnCo_2S_4$ (denoted as ZCS).

[4]S5@Pt/TiO_2 stands for Pt/TiO_2 coupled with an organic blue-colored dye (denoted as S5).

[5]CuPc/TiO_2 stands for TiO_2 supported by copper phthalocyanine (denoted as CuPc).

[6]N. A. means not available, i.e. the irradiation power was not provided.

[7]N. A. means not available, i.e. the hydrogen yield was not provided.

Table S4. Performance comparison of photocatalytic H_2 production with the state-of-the-art TiO_2-based, NIR-responsive photocatalysts reported in the literature in the recent three years.

Catalysts [co-catalyst]	Light Source	Electrolyte	Activity	Ref.
TiO_2-Au@Cu_7S_4 [none]	AM 1.5 G 100 mW/cm^2	Na_2SO_3 (0.5 M)	92.08 μmol h^{-1} g^{-1} AQY = 4.17 % (800 nm) 3.66 % (2200 nm)	This work
5.0 wt% PbS/(Pt–TiO_2) [1.0 wt% Pt]	Xenon lamp 100 mW/cm^2	Na_2S (0.35 M) and Na_2SO_3 (0.25 M)	16260 μmol h^{-1} g^{-1} AQY = 0.11 % (950nm)	(7)
[1]SA1/Pt-TiO_2 [0.75 wt % Pt]	Xenon lamp 150 mW/cm^2	Ascorbic acid (0.57 M)	451.39 μmol h^{-1} g^{-1} AQY = 1.4 % (800 nm)	(17)
[2]HPT-0.25 [1 wt % Pt]	Xenon lamp [4]N. A.	Triethanolamine (10 %)	30133 μmol h^{-1} g^{-1} AQY = 0.50 % (800 nm)	(18)
[3]Zn-tri-PcNc/TiO_2 [1 wt % Pt]	Xenon lamp [4]N. A.	Ethylenediamine tetraacetic acid disodium (10 mM)	3783 μmol h^{-1} g^{-1} AQY = 0.1 % (800 nm)	(19)

[1]SA1/Pt-TiO_2 stands for Pt-loaded TiO_2 sensitized by squaraine dye (denoted as SA1).

[2]HPT-0.25 stands for hierarchical porous TiO_2 (denoted as HPT) sensitized by 0.25 μmol PCH-001 dye.

[3]Zn-tri-PcNc/TiO_2 stands for TiO_2 sensitized by zinc phthalocyanine (denoted as Zn-tri-PcNc).

[4]N. A. means not available, i.e. the irradiation power was not provided.

Table S5. BET surface area, BJH adsorption pore volume, and average pore diameter for TiO_2-Au@Cu_2O and TiO_2-Au@Cu_7S_4.

Sample	BET surface area (m^2/g)	BJH adsorption pore volume (cm^3/g)	BJH adsorption pore diameter (nm)
TiO_2-Au@Cu_2O	17.06	0.061	40.11
TiO_2-Au@Cu_7S_4	18.19	0.072	24.52

Table S6. Comparison of photocatalytic hydrogen production over TiO_2 photocatalysts with different crystalline phases.

Catalysts [co-catalyst]	Light Source	Electrolyte	Activity	Ref.
Monoclinic TiO_2 [none]	AM 1.5 G 100 mW/cm^2	Na_2SO_3 (0.5 M)	5 μmol h^{-1} g^{-1}	This work
Anatase TiO_2 [0.5 wt% Pt]	AM 1.5 G 100 mW/cm^2	Methanol (18.519 %)	4029.4 μmol h^{-1} g^{-1}	(20)
Anatase TiO_2 [none]	Xenon lamp 100 mW/cm^2	Methanol (20 %)	5.6 μmol h^{-1} g^{-1}	(21)
Anatase TiO_2 [none]	UV light [1]N. A.	Methanol (3.125 %)	2700 μmol h^{-1} g^{-1}	(22)
Anatase TiO_2 [none]	Xenon lamp 100 mW/cm^2	Methanol (20 %)	240 μmol h^{-1} g^{-1}	(23)
Anatase TiO_2 [0.03% Pt]	Xenon lamp [1]N. A.	Methanol (20 %)	652 μmol h^{-1} g^{-1}	(24)
Anatase TiO_2 [0.5 wt% Pt]	Xenon lamp [1]N. A.	Glycerol (10%)	5059 μmol h^{-1} g^{-1}	(25)
Anatase TiO_2 [none]	Xenon lamp 100 mW/cm^2	Methanol (50 %)	22600 μmol h^{-1} g^{-1}	(26)

Rutile TiO_2 [none]	Xenon lamp 100 mW/cm^2	Methanol (20 %)	110 μmol h^{-1} g^{-1}	(23)
Rutile TiO_2 [0.03% Pt]	Xenon lamp [1]N. A.	Methanol (20 %)	526 μmol h^{-1} g^{-1}	(24)
Monoclinic TiO_2 [0.5 wt% Pt]	AM 1.5 G 100 mW/cm^2	Methanol (18.519 %)	9372.9 μmol h^{-1} g^{-1}	(20)
Monoclinic TiO_2 [none]	Xenon lamp 100 mW/cm^2	Methanol (20 %)	40.8 μmol h^{-1} g^{-1}	(21)
Monoclinic TiO_2 [0.5 wt% Pt]	Xenon lamp [1]N. A.	Glycerol (10%)	3387 μmol h^{-1}	(25)
Monoclinic TiO_2 [none]	Xenon lamp 100 mW/cm^2	Methanol (50 %)	5350 μmol h^{-1} g^{-1}	(26)
Monoclinic TiO_2 [none]	Xenon lamp (UV region) [1]N. A.	Methanol (20 %)	1505.3 μmol h^{-1} g^{-1}	(27)
Monoclinic TiO_2 [1 wt% Pt]	Xenon lamp 100 mW/cm^2	Methanol (20 %)	482 μmol h^{-1}	(28)

[1]N. A. means not available, i.e. the irradiation power was not provided.

References

(1) Jitputti, J.; Suzuki, Y.; Yoshikawa, S. Synthesis of TiO_2 Nanowires and Their Photocatalytic Activity for Hydrogen Evolution. *Catal. Commun.* **2008**, *9* (6), 1265-1271. DOI: 10.1016/j.catcom.2007.11.016

(2) Ji, X.; Song, X.; Li, J.; Bai, Y.; Yang, W.; Peng, X. Size Control of Gold Nanocrystals in Citrate Reduction: The Third Role of Citrate. *J. Am. Chem. Soc.* **2007**, *129* (45), 13939-13948. DOI: 10.1021/ja074447k

(3) Fang, M.-J.; Lin, Y.-C.; Jan, J.-Y.; Lai, T.-H.; Hsieh, P.-Y.; Kuo, M.-Y.; Chiu, Y.-H.; Tsao, C.-W.; Chen, Y.-A.; Wang, Y.-T.; et al. Au@Cu_2O Core@Shell Nanocrystals as Sustainable Catalysts for Efficient Hydrogen Production from Ammonia Borane. *Appl. Catal. B Environ.* **2023**, *324*, 122198. DOI: 10.1016/j.apcatb.2022.122198

(4) Li, J.-M.; Tsao, C.-W.; Fang, M.-J.; Chen, C.-C.; Liu, C.-W.; Hsu, Y.-J. TiO_2-Au-Cu_2O Photocathodes: Au-Mediated Z-Scheme Charge Transfer for Efficient Solar-Driven Photoelectrochemical Reduction. *ACS Appl. Nano Mater.* **2018**, *1* (12), 6843-6853. DOI: 10.1021/acsanm.8b01678

(5) Wang, H.; Jiang, H.; Huo, P.; Filip Edelmannová, M.; Čapek, L.; Kočí, K. Hydrogen Production from Methanol-Water Mixture over NiO/TiO_2 Nanorods Structure Photocatalysts. *J. Environ. Chem. Eng.* **2022**, *10* (1), 106908. DOI: 10.1016/j.jece.2021.106908

(6) Gogoi, D.; Namdeo, A.; Golder, A. K.; Peela, N. R. Ag-Doped TiO_2 Photocatalysts with Effective Charge Transfer for Highly Efficient Hydrogen Production through Water Splitting. *Int. J. Hydrogen Energy* **2020**, *45* (4), 2729-2744. DOI: 10.1016/j.ijhydene.2019.11.127

(7) Wang, J.; Chen, S.; Liu, D.; Chen, C.; Li, R.; Peng, T. Fabrication of PbS Nanocrystal-Sensitized Ultrafine TiO_2 Nanotubes for Efficient and Unusual Broadband-Light-Driven Hydrogen Production. *Mater. Today Chem.* **2020**, *17*, 100310. DOI: 10.1016/j.mtchem.2020.100310

(8) Lv, S.; Wang, Y.; Zhou, Y.; Liu, Q.; Song, C.; Wang, D. Oxygen Vacancy Stimulated Direct Z-Scheme of Mesoporous Cu_2O/TiO_2 for Enhanced Photocatalytic Hydrogen Production from Water and Seawater. *J. Alloys Compd.* **2021**, *868*, 159144. DOI: 10.1016/j.jallcom.2021.159144

(9) Qiu, Z.-s.; Zhou, T.; Ma, Y.-w.; Ma, Y.-x.; Lv, T.-p.; Zhao, J.-h.; Zhang, J.; Zhang, Y.-m.; Liu, Q.-j. Single-Atom Pd Supported on TiO_2 for the Photocatalytic Production of Hydrogen. *ACS Appl. Nano Mater.* **2023**, *6* (24), 23524-23531. DOI: 10.1021/acsanm.3c04909

(10) Rej, S.; Hejazi, S. M. H.; Badura, Z.; Zoppellaro, G.; Kalytchuk, S.; Kment, Š.; Fornasiero, P.; Naldoni, A. Light-Induced Defect Formation and Pt Single Atoms Synergistically Boost Photocatalytic H_2 Production in 2D TiO_2-Bronze Nanosheets.

ACS Sustain. Chem. Eng. **2022**, *10* (51), 17286-17296. DOI: 10.1021/acssuschemeng.2c05708
(11) Makino, K.; I, T.; Kubo, Y. A Benzofuran[b]-Fused BODIPY as an Efficient Sensitizer for Photocatalytic Hydrogen Production. *Sustain. Energy Fuels* **2021**, *5* (14), 3676-3686. DOI: 10.1039/D1SE00387A
(12) Khojiev, S.; Hojiyeva, G.; Tursunkulov, O.; Chen, D.; S. H. Mohamed, H.; Kholikov, A.; Akbarov, K.; Liu, J.; Li, Y. Red Emissive Upconversion Carbon Quantum Dots Modifying TiO_2 for Enhanced Photocatalytic Performance. *ACS Appl. Nano Mater.* **2023**, *6* (16), 14669-14679. DOI: 10.1021/acsanm.3c02012
(13) Nagoor Meeran, M.; Haridharan, N.; Shkir, M.; Algarni, H.; Reddy Minnam Reddy, V. Rationally Designed 1D $CdS/TiO_2@Ti_3C_2$ Multi-Components Nanocomposites for Enhanced Visible Light Photocatalytic Hydrogen Production. *Chem. Phys. Lett.* **2022**, *809*, 140150. DOI: 10.1016/j.cplett.2022.140150
(14) Dai, X.; Feng, S.; Wu, W.; Zhou, Y.; Ye, Z.; Cao, X.; Wang, Y.; Yang, C. Photocatalytic Hydrogen Evolution and Antibiotic Degradation by S-Scheme $ZnCo_2S_4/TiO_2$. *Int. J. Hydrogen Energy* **2022**, *47* (60), 25104-25116. DOI: 10.1016/j.ijhydene.2022.05.269
(15) Ding, H.; Xu, M.; Zhang, S.; Yu, F.; Kong, K.; Shen, Z.; Hua, J. Organic Blue-Colored D-A-π-A Dye-Sensitized TiO_2 for Efficient and Stable Photocatalytic Hydrogen Evolution under Visible/Near-Infrared-Light Irradiation. *Renew. Energy* **2020**, *155*, 1051-1059. DOI: 10.1016/j.renene.2020.04.009
(16) Moon, H. S.; Yong, K. Noble-Metal Free Photocatalytic Hydrogen Generation of $CuPc/TiO_2$ Nanoparticles under Visible-Light Irradiation. *Appl. Surf. Sci.* **2020**, *530*, 147215. DOI: 10.1016/j.apsusc.2020.147215
(17) Shen, X.-F.; Watanabe, M.; Takagaki, A.; Song, J. T.; Abe, T.; Kawaguchi, D.; Tanaka, K.; Ishihara, T. Pyridyl Anchoring Squaraine as a Near-Infrared Dye Sensitizer for Effective Sensitized Hydrogen Production over a Titanium Dioxide Photocatalyst in Water Medium. *Appl. Phys. A* **2022**, *129* (1), 28. DOI: 10.1007/s00339-022-06281-7
(18) Tiwari, A.; Krishna, N. V.; Giribabu, L.; Pal, U. Hierarchical Porous TiO_2 Embedded Unsymmetrical Zinc–Phthalocyanine Sensitizer for Visible-Light-Induced Photocatalytic H_2 Production. *J. Phys. Chem. C* **2018**, *122* (1), 495-502. DOI: 10.1021/acs.jpcc.7b09759
(19) Zhang, X.; Yu, L.; Zhuang, C.; Peng, T.; Li, R.; Li, X. Highly Efficient Visible/Near-IR-Light-Driven Photocatalytic H_2 Production over Asymmetric Phthalocyanine-Sensitized TiO_2. *RSC Adv.* **2013**, *3* (34), 14363-14370. DOI: 10.1039/C3RA41975D
(20) Li, P.; Cao, Q.; Zheng, D.; Alshehri, A. A.; Alghamidi, Y. G.; Alzahrani, K. A.;

Kim, M.; Hou, J.; Lai, L.; Yamauchi, Y.; et al. Synthesis of Mesoporous TiO_2-B Nanobelts with Highly Crystalized Walls toward Efficient H_2 Evolution. *Nanomater.* **2019**, *9* (7), 919. DOI: 10.3390/nano9070919
(21) Zhang, Y.; Xing, Z.; Liu, X.; Li, Z.; Wu, X.; Jiang, J.; Li, M.; Zhu, Q.; Zhou, W. Ti^{3+} Self-Doped Blue TiO_2(B) Single-Crystalline Nanorods for Efficient Solar-Driven Photocatalytic Performance. *ACS Appl. Mater. Interfaces.* **2016**, *8* (40), 26851-26859. DOI: 10.1021/acsami.6b09061
(22) Li, F.; Huang, Y.; Peng, H.; Cao, Y.; Niu, Y. Preparation and Photocatalytic Water Splitting Hydrogen Production of Titanium Dioxide Nanosheets. *Int. J. Photoenergy* **2020**, *2020* (1), 3617312. DOI: 10.1155/2020/3617312
(23) Ding, L.; Yang, S.; Liang, Z.; Qian, X.; Chen, X.; Cui, H.; Tian, J. TiO_2 Nanobelts with Anatase/Rutile Heterophase Junctions for Highly Efficient Photocatalytic Overall Water Splitting. *J. Colloid Interface Sci.* **2020**, *567,* 181-189. DOI: 10.1016/j.jcis.2020.02.014
(24) Wang, Q.; Lian, J.; Bai, Y.; Hui, J.; Zhong, J.; Li, J.; An, N.; Yu, J.; Wang, F. Photocatalytic Activity of Hydrogen Production from Water over TiO_2 with Different Crystal Structures. *Mater. Sci. Semicond. Process.* **2015**, *40*, 418-423. DOI: 10.1016/j.mssp.2015.06.089
(25) Qiu, Y.; Ouyang, F. Fabrication of TiO_2 Hierarchical Architecture Assembled by Nanowires with Anatase/TiO_2(B) Phase-Junctions for Efficient Photocatalytic Hydrogen Production. *Appl. Surf. Sci.* **2017**, *403*, 691-698. DOI: 10.1016/j.apsusc.2017.01.255
(26) Cai, J.; Wang, Y.; Zhu, Y.; Wu, M.; Zhang, H.; Li, X.; Jiang, Z.; Meng, M. In Situ Formation of Disorder-Engineered TiO_2(B)-Anatase Heterophase Junction for Enhanced Photocatalytic Hydrogen Evolution. *ACS Appl. Mater. Interfaces.* **2015**, *7* (45), 24987-24992. DOI: 10.1021/acsami.5b07318
(27) Xu, M.; Khan, M. S.; Fazil, P.; Ateeq, M. Effect of Calcination Temperature on the Photocatalytic H_2 Evolution of Bronze Phase Monoclinic TiO_2(B) Nanosheets. *J. Chem. Soc. Pak.* **2022**, *44* (3), 223-223. DOI: 10.52568/001035/JCSP/44.03.2022
(28) Li, Q.; Wu, P.; Huang, Y.; Chen, P.; Wu, K.; Wu, J.; Luo, Y.; Wang, L.; Yang, S.; Liu, Z.; et al. A Novel Lattice-Embedded ZnO@TiO_2(B) Nanoflowers Promotes Photocatalytic Production of H_2. *Int. J. Hydrogen Energy* **2022**, *47* (35), 15641-15654. DOI: 10.1016/j.ijhydene.2022.03.091
